# Low-dose EGFR inhibition unlocks ferroptosis susceptibility to sensitize chemotherapy in EGFR-high triple-negative breast cancer

**DOI:** 10.1016/j.jbc.2026.113185

**Published:** 2026-05-25

**Authors:** Xiaoxi Li, Ling Liu, Lingli Luo, Minyao Deng, Yong Jiang, Weijun Ren, Hui Qian

**Affiliations:** Department of Laboratory Medicine, School of Medicine, Jiangsu University, Zhenjiang, Jiangsu, China

**Keywords:** chemosensitization, EGFR, PROTAC, ferroptosis, triple-negative breast cancer

## Abstract

Triple-negative breast cancer (TNBC) is a highly heterogeneous subtype with a poor prognosis and limited therapeutics, for which metastasis is a primary driver. In this study, we explore the function of EGFR in breast cancer progression and the mechanistic basis of EGFR-targeted therapies, focusing on kinase inhibitors and protein degraders. In a murine breast cancer model, EGFR knockout exhibited minimal impact on primary tumor growth but significantly suppressed metastasis, supporting the clinical association between high EGFR expression and poor prognosis. At high doses, EGFR degraders exert their antitumor effect through kinase inhibition, not degradation, whereas low-dose EGFR inhibition has limited anti-proliferative activity. Gene-drug interaction screening identified gemcitabine (GEM) and decitabine (DAC) as agents interacting with EGFR, a finding confirmed by the observed resistance to these drugs in TNBC cell lines with high EGFR expression. Both low-dose EGFR degraders and inhibitors chemosensitize cells to GEM/DAC *in vitro*, with the EGFR degrader plus GEM combination exhibiting an enhanced inhibitory effect in a lung colonization model. Mechanistically, both low-dose EGFR degraders and inhibitors elevate ROS levels and induce lipid peroxidation, thereby sensitizing cancer cells to GEM/DAC-triggered ferroptosis. In summary, this study demonstrates that low-dose EGFR targeting creates a susceptible state for ferroptosis in tumor cells and, consequently, defines a combination therapy strategy of EGFR-targeted agents with chemotherapy for TNBC. Our results provide a foundation for the clinical translation of low-dose EGFR-targeting strategies and their rational combination with other agents.

EGFR, frequently dysregulated through gain-of-function mutations in multiple cancer types, represents a highly promising therapeutic target. In addition to gain-of-function mutations, aberrant DNA methylation serves as a critical mechanism underlying the dysregulation of oncogenic driver gene expression. Recent studies have revealed that EGFR hypomethylation is prevalent across multiple tumor types (1). However, current EGFR-targeted interventions, including monoclonal antibodies and small-molecule inhibitors, have demonstrated limited efficacy across tumor types beyond lung cancer and often induce adaptive resistance. The clinical efficacy of tyrosine kinase inhibitors (TKIs) targeting EGFR is quite limited (2). Multiple mechanisms, including impaired protein degradation (3), can lead to tumors developing acquired resistance to TKIs. Targeted protein degradation (TPD) emerges as a novel antitumor strategy (4, 5). It directly triggers the degradation of target proteins, offering a highly anticipated solution to circumvent acquired resistance to kinase inhibitors (6, 7).

Metastatic breast cancer, also known as Stage IV breast cancer, refers to the dissemination of cancer cells from the breast to other parts of the body, with common sites of metastasis including bones, brain, lungs, liver, and others. Approximately 30% of patients with early-stage breast cancer will develop metastatic breast cancer, which currently cannot be predicted nor cured. The average time to first distant metastasis for hormone receptor-positive (HR+) patients is 1 to 2 years, while for hormone receptor-negative (HR-) patients, it occurs within one year. Among HR-subtypes, HER2-EGFR + has the fastest metastasis rate, followed by HER2+, and HER2-EGFR-has the slowest (8, 9). Hormone therapy serves as a therapeutic option for patients with HR + breast cancer, while HER2-targeted therapy is applicable to those with HER2+ disease. Nevertheless, a specific treatment regimen for TNBC remains elusive (10).

The evaluation of drug efficacy is the cornerstone of antitumor drug development, aimed at predicting the effectiveness of drugs. Yet, the alarming high failure rate in clinical trials clearly indicates that the existing preclinical efficacy evaluation framework is inadequate and in need of refinement (11, 12). We hypothesize that how well preclinical cancer models match clinical scenarios real-world clinical scenarios and the drug dosage are two key factors for improving the precision of efficacy evaluation predictions. Most antitumor drug efficacy evaluation models now, like the nude mouse subcutaneous tumor model or organoid models, use tumor growth inhibition as the evaluation standard. But in clinics, systemic metastasis is the main reason cancer patients die. So, evaluating if a drug can prevent or stop tumor metastasis, not just tumor growth, is crucial for improving efficacy evaluation predictions (13). In addition, an undeniable fact is that in the majority of preclinical efficacy evaluation models, the drug dosage applied is excessively high and bears no resemblance to the effective dosage in the human body (14). For instance, the primary effect of most kinase inhibitors is to inhibit tumor cell proliferation, while an overly high dosage can induce cell apoptosis. Therefore, evaluating a drug's ability to prevent or inhibit tumor metastasis at a proper dosage is the main way to improve preclinical efficacy evaluation, facilitate clinical translation, and boost the success rate of clinical trials.

In this study, we employed the 4T1 orthotopic transplant and resection (4T1-OtR) model (13) to assess the dependency of tumor growth and metastasis on EGFR. We evaluated the long-term inhibitory effects of EGFR inhibitors and EGFR degraders on tumor cells, as well as the impact of external nutrients. Additionally, we compared the biological effects of EGFR inhibitors and EGFR degraders at their effective inhibitory doses and observed that both agents promoted lipid metabolism. Furthermore, we developed a GFP-competition assay to analysis drug-interaction and identified gemcitabine/decitabine (GEM/DAC) as interacting drugs with EGFR. We then examined the antitumor activity of the combination of EGFR intervention and chemotherapy both *in vitro* and *in vivo*. These findings offer crucial insights into the potential of EGFR as a therapeutic target and the potential application of its drug combinations in preventing breast cancer metastasis.

## Results

### EGFR overexpression is linked to a worse prognosis in breast cancer, especially in HR-subtype

**Figure 1:**
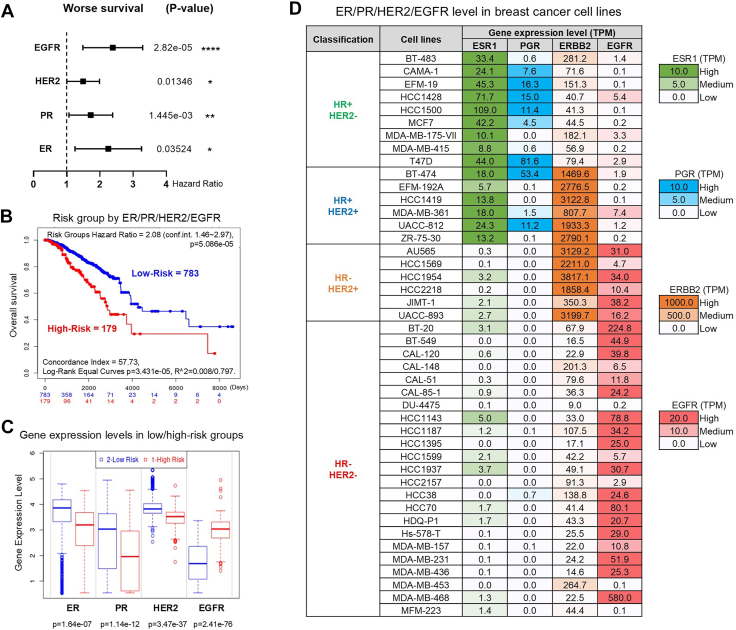
**The clinical relevance of EGFR expression level in breast cancer prognosis and its relationship with distinct subtypes.***A*, Forest plot for the prognostic performance of ER, PR, HER2, and EGFR. *p*-Value of the log-rank test were shown. A human BRCA dataset (TCGA-BRCA, n = 962) was chosen to survival analysis. The hazard ratio (HR), confidence interval, *p*-Value in forest plot were obtained from the SurvExpress program. *B* and *C*, survival curve of BRCA patients (*B*) and the expression level of ER, PR, HER2, EGFR (*C*) in low-risk and high-risk groups. The prognostic index (PI) was calculated by the joint expression level of the eight genes and the Cox model to generate the risk groups. The optimization algorithm was applied in risk grouping by the SurvExpress program. *D*. Gene expression level of ER, PR, HER2, EGFR in breast cancer cell lines. Gene expression level is evaluated by TPM. TPM, Transcripts Per Kilobase per Million mapped reads.

Extensive evidence indicates that high EGFR protein expression promotes malignant progression and is associated with poor prognosis in breast cancer (15, 16, 17, 18, 19, 20). However, existing prognostic data are largely derived from IHC-based protein detection. Given the quantitative resolution of mRNA expression quantified by RNA-seq, we systematically examined the associations between EGFR transcript levels, clinical outcomes, and the expression of standard biomarker genes (ER, PR, and HER2). We found that EGFR expression levels were strongly linked to breast cancer prognosis (*p* = 2.82 × 10^−5^), much better than ER, PR, and HER2 (Fig. 1*A*). Next, combining the expression levels of these four genes further enhanced the accuracy of stratifying patients into high-risk and low-risk groups (Fig. 1*B*). Moreover, EGFR expression levels were significantly higher in the high-risk group than in the low-risk group (*p* = 2.41 × 10^−76^) (Fig. 1*C*), suggesting that EGFR expression levels possess strong diagnostic efficacy.

To evaluate the subtype-specific expression pattern of EGFR, we interrogated transcriptomic data of breast cancer cell lines from the DepMap portal. The results showed that EGFR and HR status were mutually exclusive. EGFR was predominantly expressed in HR-cell lines and absent in HR + cell lines (Fig. 1*D*), implying that EGFR serves as a specific marker for HR-breast cancer.

### EGFR represents a potential therapeutic target for intervening in breast cancer metastasis

**Figure 2:**
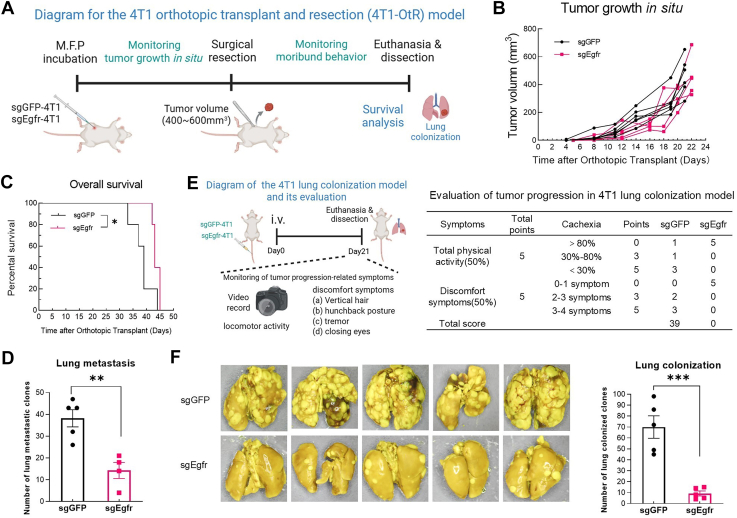
**Evaluating EGFR dependency in malignant progression of breast cancer in the 4T1-OtR model.***A*, diagram of the experimental protocol for the 4T1-OtR model. 4T1 cells were injected into the mammary fat pad (M.F.P.), and tumor growth was monitored. Upon reaching a tumor volume within the range of 400 to 600 mm^3^, the primary tumor was surgically removed. The disease progression of the mice was monitored. When the mice showed signs of impending death, the mice were euthanized, and the tumor metastasis was examined through autopsy. *B*, tumor volume of orthotopic tumors. n(sgGFP) = 6, n(sgEgfr) = 5. *C*, overall survival time of mice treated with sgGFP and sgEgfr. Log-rank (Mantel–Cox) tests were used to test the significance. n(sgGFP) = 5, n(sgEgfr) = 5. *D*, quantification of tumor lung metastasis. Unpaired t-tests were used to test the significance. n(sgGFP) = 5, n(sgEgfr) = 4. *E*, diagram of the 4T1 lung colonization model and phenotypic evaluation of disease progression. *F*. Images and quantification of tumor lung colonization. The number of colonized tumor clones were measured on both front and back surfaces of the lungs. Here, only front-view images are presented. Unpaired t-tests were used to test the significance.

Given the strong association between EGFR levels and breast cancer prognosis, we hypothesize that EGFR may play an indispensable role in breast cancer metastasis. To evaluate the dependency of breast cancer metastasis on EGFR, we simulated the clinical scenario of breast cancer disease progression. By performing orthotopic transplantation of 4T1 breast cancer cells and subsequently surgically removing tumours once they reached a predefined size, we were able to assess the effects of EGFR on both local tumor growth and distant metastasis in breast cancer (Fig. 2*A*). More significantly, the surgical removal of the primary tumor can prolong the survival time of mice, which, in turn, enables the tumor to disseminate to a greater number of sites.

The results demonstrate that, in comparison to the control group, EGFR knockout did not significantly impact the growth of orthotopic tumors (Figs. 2*B* and S1*A*). However, it led to a prolonged overall survival time in mice (Fig. 2*C*). Upon final autopsy, the control group exhibited lung metastasis and pleural implantation as the primary manifestations of tumor recurrence, accompanied by lumbar vertebral invasion. In contrast, the EGFR-knockout group showed a reduction in both lung metastasis and pleural implantation (Figs. 2*D* and S1, *B* and *C*). Nevertheless, there was an emergence of visceral organ involvement, including the kidneys and liver, along with thoracic vertebral invasion; lumbar vertebral invasion was not observed (Fig. S1, *B* and *C*). These findings suggest that EGFR plays a crucial role in tumor metastasis rather than tumor growth. We hypothesize that EGFR can influence tumor cell colonization, thereby impacting tumor progression, treatment outcomes, and prognosis.

The process from the development of orthotopic tumors to the formation of distant metastatic lesions involves a multi-step and complex biological cascade. To evaluate the process of tumor colonization in the lungs, we established a 4T1 lung colonization model using the tail vein injection method (Fig. 2*E*). The time point at which a specific group of mice exhibited distinct phenotypic manifestations of disease progression was designated as the experimental endpoint. The disease-progression phenotype was determined by scoring the mice based on their motor ability and discomfort symptoms (Fig. 2*E* and Movies S1 and S2). The results revealed that EGFR knockout significantly reduced the number of tumor colonies in the lungs, establishing a key role for EGFR in the lung colonization process and underscoring its potential as a therapeutic target for preventing breast cancer metastasis.

### The antitumor efficacy of EGFR kinase inhibitors can be antagonized by compensatory proliferative signals

We then re-analyzed the inhibitory effects of EGFR inhibitors and genetic deletion on tumor cell proliferation. It should be noted that EGFR exerts its functions not only through its kinase activity but also *via* kinase-independent mechanisms, such as interacting with other signaling proteins. EGFR inhibitors can only block the kinase activity without fully abrogating all EGFR functions. Many studies indicate that excessively high inhibitor concentrations can confound efficacy evaluation (14). Additionally, we would like to highlight that in this study, the drug treatment duration was extended from the conventional 1 to 3 days to 5 days, with correspondingly lower doses applied. We believe that prolonged, low-dose treatment represents a critical refinement for more accurate drug efficacy evaluation.

To dissect the specific contribution of EGFR kinase inhibition to the observed antitumor effects, we utilized an EGFR/HER2 dual-target inhibitor as a reference control. EGFR-monospecific inhibitors (Erlotinib and Gefitinib) and EGFR/HER2-dual inhibitors (Lapatinib and Sapitinib), which have comprehensive drug response data in the GDSC database, were selected for subsequent analysis. The anti-EGFR activity of the dual EGFR/HER2 inhibitor was no greater than that of the selective EGFR inhibitor. According to public data, in cell-free assays, Lapatinib exhibits IC50 values of 10.2 nM for EGFR and 9.8 nM for ErbB2, while Erlotinib shows an IC50 of 2 nM for EGFR.

**Figure 3:**
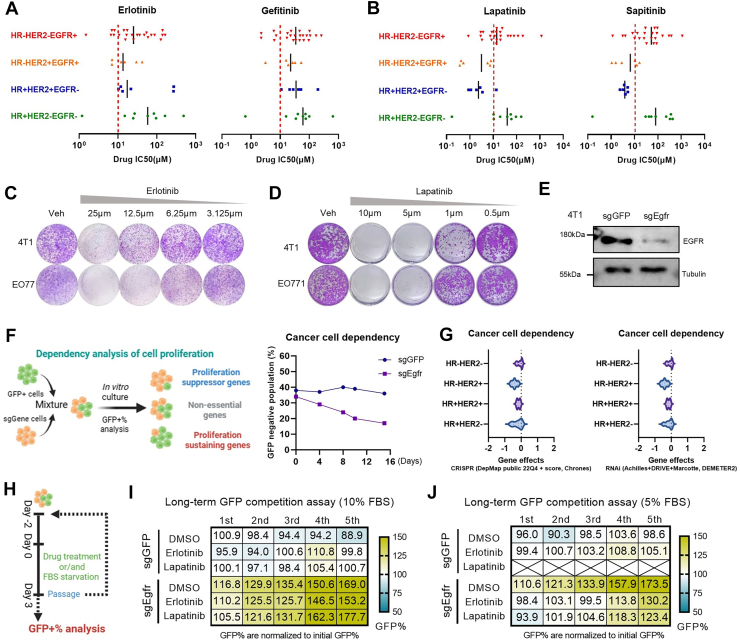
**Inhibitory effects of EGFR inhibition and genetic ablation on tumor cell proliferation.***A* and *B*, the IC_50_ of EGFR-targeting inhibitors (*A*) and EGFR/HER2-targeting inhibitors (*B*) in breast cancer cell lines. *C* and *D*. Inhibitory effects of Erlotinib and Lapatinib on 4T1 and EO771 cell lines. *E*. Western blot of EGFR knockout in 4T1 cell line. *F*. Diagram illustrating the analysis of cell proliferation dependency. The proportional changes in GFP^+^ and GFP^-^ cell populations during long-term *in vitro* culture. GFP^-^ cells undergo EGFR knockout, and GFP^+^ cells maintain normal EGFR. *G*. The gene effects of EGFR on cancer cell dependency in four subtypes of breast cancer cell lines. *H*. Diagram depicting the analysis of cell proliferation dependency under long-term FBS-starvation conditions. *I* and *J*. Long-term inhibitory impacts of EGFR inhibitors on GFP^-^ cells under normal FBS (*I*) and FBS-starved (*J*) conditions.

We designated 10 μm as the threshold for excessive/resistant/ineffective doses. It should also be noted that most HER2-negative cells still express HER2 (Fig. 1*D*), but at lower levels than amplified HER2-positive lines. Results revealed that, for HER2^+^ breast cancer cell lines, EGFR/HER2-dual inhibitors exhibited superior inhibitory efficacy than EGFR-monospecific inhibitors (Fig. 3, *A* and *B*). In contrast, for HR^-^HER2^-^EGFR^+^ breast cancer cell lines, only a small proportion of cells were susceptible to EGFR-monospecific inhibitors, whereas a larger number of cells could be inhibited by the EGFR/HER2-dual inhibitor Lapatinib (Fig. 3, *A* and *B*). Subsequently, we validated the long-term inhibitory effects of Erlotinib and Lapatinib in 4T1 and EO771. The results demonstrated that the effective inhibitory dose of Erlotinib was indeed significantly higher than that of Lapatinib (Fig. 3, *C* and *D*).

These findings indicate that most HR-HER2- EGFR + breast cancer cell lines show a higher reliance on HER2 kinase activity over EGFR kinase activity for their proliferation and survival.

To investigate the kinase-independent roles of EGFR in cell proliferation *in vitro*, we established a co-culture system of EGFR-knockout and GFP-positive cells and longitudinally tracked the GFP ratio over time. Our experimental data revealed that EGFR knockout modestly reduced the GFP-negative cell population, implying a weak anti-proliferative effect (Fig. 3, *E* and *F*). This observation aligns with DepMap database analysis, showing that genetic depletion of EGFR exerts only minor growth effects across human breast cancer cell lines, including the TNBC subtype (Fig. 3*G*). These results indicate that neither pharmacological inhibition nor genetic knockout of EGFR effectively suppresses cell proliferation *in vitro*.

We therefore hypothesize that pro-proliferation factors in the *in vitro* culture or *in vivo* niche, such as cytokines, may sustain the proliferation of EGFR-knockout cells by activating alternative signaling pathways. To validate the influence of the culture conditions, we performed long-term drug treatments involving serum starvation and EGFR inhibitors, building upon the GFP-dependency analysis experiment (Fig. 3*H*). The data demonstrated that in a nutrient-abundant setting (10% FBS) (Fig. 3*I*), EGFR knockout caused a gradual inhibition of cell proliferation, and the EGFR inhibitors had no impact on the change in the GFP ratio. That is, the proliferation of wild-type EGFR control cells remained unaffected by EGFR inhibitors. In contrast, in a nutrient-limited condition (5% FBS) (Fig. 3*J*), EGFR inhibitor treatment markedly offset the decline in the GFP ratio resulting from EGFR knockout, indicating that EGFR inhibitors exhibited a notable inhibitory effect on the proliferation of wild-type EGFR control cells. These findings suggest that standard culture media contain sufficient mitogenic signals to compensate for EGFR loss. This compensatory mechanism plays a crucial role in attenuating the inhibitory efficacy of EGFR inhibitors.

### Antitumor effects of EGFR degraders arise from kinase inhibition

Targeted protein degradation represents a cutting-edge therapeutic strategy that operates through a mechanism fundamentally distinct from that of kinase inhibitors. By inducing the degradation of target proteins, targeted protein degradation methods not only ablate its kinase-dependent functions but also disrupts its kinase-independent scaffolding roles. Moreover, targeted protein degraders are designed to overcome acquired resistance mutations that arise in response to kinase inhibitor therapy. Consequently, protein degraders have the potential to elicit antitumor responses that differ significantly from those achieved through kinase inhibition alone.

**Figure 4:**
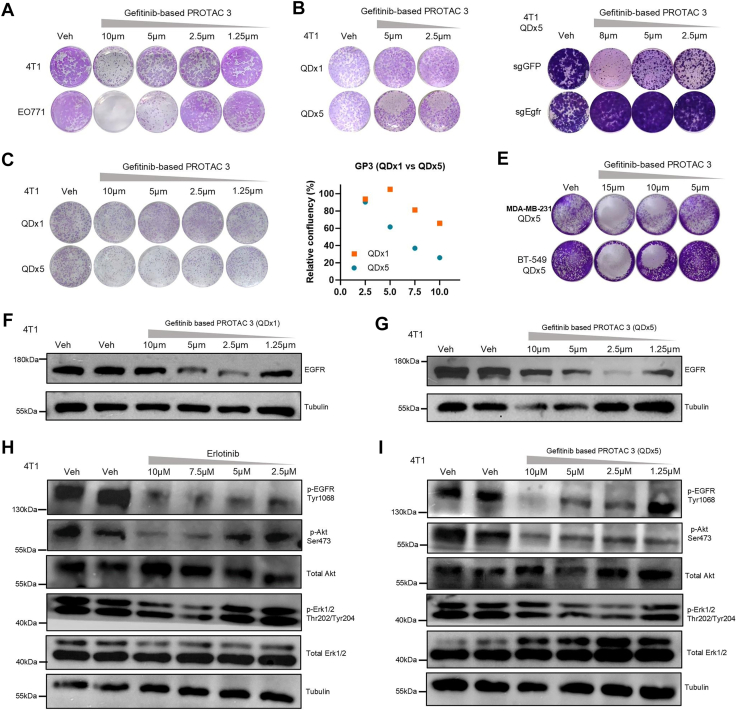
**Inhibitory effects of EGFR degraders on tumor cell proliferation.***A*, the inhibitory efficacy of a single-dose GP3 administration on 4T1 and EO771 cells. *B*, comparative effective of a single-dose (QDx1) and continuous (QDx5) administration of GP3 on the proliferation of 4T1 cells. QDx1: 1-day treatment; QDx5: 5-days continuous treatment. *C*, impact of single-dose *versus* continuous GP3 administration on 4T1 colony formation and confluency, measured *via* crystal violet staining. *D*, inhibitory effect of continuous GP3 treatment on sgGFP/sgEgfr 4T1 cells. *E*. Inhibitory effect of continuous GP3 treatment on human TNBC cell lines. *F* and *G*, Western blot analysis of the effects of a single-dose and continuous administration of GP3 on the induction of EGFR degradation. *H* and *I*. Western blot analysis of the effects of Erlotinib (*F*) and GP3 (*G*) on the phosphorylation of EGFR and its downstream targets.

With this in mind, we examined the inhibitory effects of the EGFR degrader Gefitinib-based PROTAC 3 (GP3), a proteolysis-targeting chimera (PROTAC) that incorporates the EGFR inhibitor Gefitinib. The results showed that GP3 could inhibit the proliferation of 4T1 and EO771 cells (Fig. 4*A*). Given GP3's poor stability, we postulated that continuous dosing could boost its inhibitory effects on tumor cells. We compared the efficacy of GP3 in inhibiting tumor cell proliferation between a single-dose treatment and a 5-days continuous treatment regimen. The results showed the 5-days treatment had no obvious inhibitory effect on tumor cell proliferation overall but did reduce cell confluence (Fig. 4*B*). In the clone formation assay, the 5-days treatment exhibited a more significant inhibitory effect compared to the single-dose treatment (Fig. 4*C*). These results imply continuous treatment boosts GP3's antitumor efficacy. We also tested GP3 in sgEGFR cells, which showed resistance to this EGFR degrader (Fig. 4*D*), thus further corroborating the on-target activity of the PROTAC molecule. Furthermore, we evaluated the anti-tumor efficacy of continuous GP3 treatment in two human TNBC cell lines, MDA-MB-231 and BT-549. In both cell lines, continuous GP3 treatment exhibited anti-proliferative activity (Fig. 4*E*).

Given the widely reported hook effect associated with PROTACs (6, 21), we analyzed the EGFR protein-degrading effects of single-dose and continuous GP3 treatment. The results demonstrated that both single-dose and continuous treatments of GP3 on 4T1 cells exhibited a distinct and similar hook effect. The most pronounced EGFR protein degradation was observed at a dose of 2.5 μm for both treatment modalities (Fig. 4, *F* and *G*).

Paradoxically, while the efficiency of target protein degradation declines at higher PROTAC concentrations, the anti-proliferative effect is enhanced. This clear dissociation strongly suggests that GP3-induced EGFR degradation is not the primary mechanism responsible for the inhibition of tumor cell proliferation. We therefore propose that the bifunctional PROTAC GP3 mediates its effects through both kinase inhibition and target degradation. The primary mechanism shifts from efficient target protein degradation at therapeutic doses to potent kinase inhibition at higher concentrations. As hypothesized, high-dose GP3—without inducing target degradation—potently suppressed phosphorylation of EGFR and downstream AKT, similar to erlotinib (Fig. 4, *H* and *I*). These results thereby confirm that the antiproliferative activity at high concentrations is driven by kinase inhibition.

### EGFR intervention remodels lipid metabolic homeostasis in tumor cells

**Figure 5:**
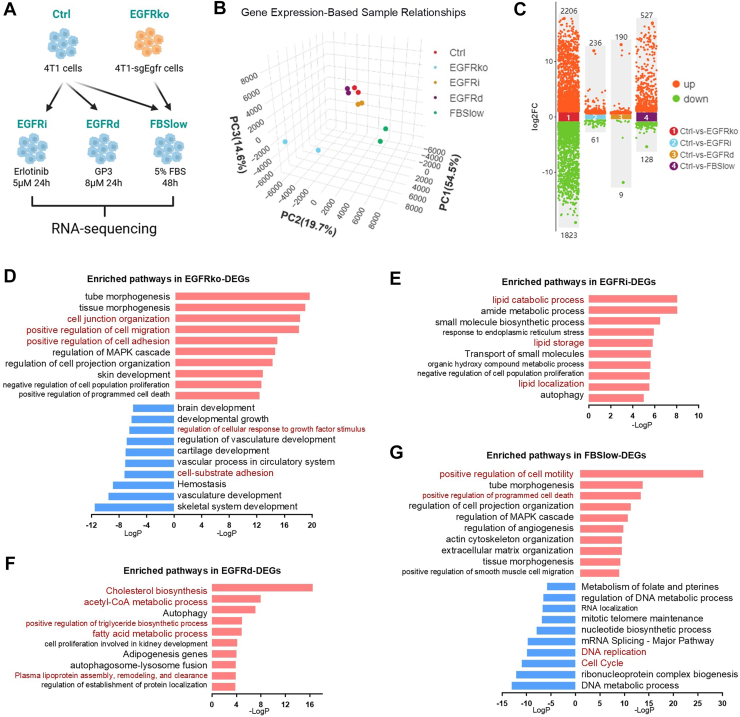
**The impact of distinct targeted EGFR approaches and low-serum conditions on the transcriptome.***A*, schematic of sequencing samples and treatments. *B*, principal component analysis (PCA) of sample relationships. *C*, differentially expressed genes (DEGs) across multiple groups. For EGFR knockout and low-serum conditions, genes with FDR< 0.05 and |fold change| ≥ 2 were considered DEGs. For EGFR inhibitor and degrader treatments, genes with FDR < 0.05 and |fold change| ≥ 1.5 were regarded as DEGs. *D*–*G*, enriched signaling pathways of DEGs resulting from EGFR knockout (*D*), EGFR inhibitor treatment (*E*), EGFR degrader treatment (*F*), and low-serum conditions (*G*).

To investigate the initial effects of EGFR degraders and inhibitors on tumor cells, we administered effective doses of these drugs to the cells and performed transcriptome analysis. EGFR-knockout cells and cells treated with low-serum conditions were included as control groups (Fig. 5*A*). Principal component analysis (PCA) revealed that EGFR knockout and low-serum conditions had a profound impact on the tumor cell transcriptome. In contrast, the effects of EGFR degraders and inhibitors on the transcriptome were relatively minor (Fig. 5*B*).

An analysis of differentially expressed genes (DEGs) showed that EGFR degraders and inhibitors had a limited influence on gene expression. In contrast, EGFR knockout triggered a massive up- and down-regulation of genes, indicating a complete reprogramming of the cell's gene expression landscape. Low-serum conditions, in particular, caused a notable increase in gene expression (Fig. 5*C*).

Pathway enrichment analysis showed that EGFR knockout mainly resulted in the up-regulation of genes associated with cell adhesion and migration and the downregulation of genes related to cell-substrate adhesion (Fig. 5*D*). This implies that changes in the surface adhesome of tumor cells could be a potential cause for the altered lung colonization ability of these cells. Additionally, the downregulation of the regulation of cellular response to growth factor stimulation suggests that EGFR-knockout cells have a reduced dependence on external nutrients.

Surprisingly, EGFR degraders and inhibitors mainly induced the upregulation of genes associated with lipid metabolism (Fig. 5, *E* and *F*). This highlights that the main outcome of pharmacological EGFR intervention is to boost the lipid metabolic activity of tumor cells, which is distinct from the effects of EGFR knockout. Low-serum conditions mainly led to the upregulation of apoptosis-related genes and the downregulation of genes involved in DNA replication and the cell cycle (Fig. 5*G*), suggesting an effect of inducing apoptosis and inhibiting cell proliferation.

To conclude, our research reveals a key insight: the main biological outcomes of both pharmacological EGFR intervention and genetic EGFR knockout are not driven by the induction of apoptosis or the suppression of cell proliferation. For pharmacological EGFR intervention, its core effect is to reestablish the balance of lipid metabolism within tumor cells. On the other hand, genetic EGFR knockout primarily acts by altering the composition of cell-surface adhesome proteins, which in turn affects the behavior of tumor cells, such as their adhesion, migration, and colonization capabilities.

### GEM, DAC, and DOX are EGFR-interacting antitumor drugs

Given that the effects of both pharmacological and genetic interventions targeting EGFR lie in reshaping the state of tumor cells rather than inhibiting their proliferation, the novel homeostatic state induced by these interventions may alter the sensitivity of tumor cells to particular antitumor drugs. Consequently, this understanding can serve as a valuable basis for tailoring tumor treatment strategies to individual patients, paving the way for more effective personalized medicine.

**Figure 6:**
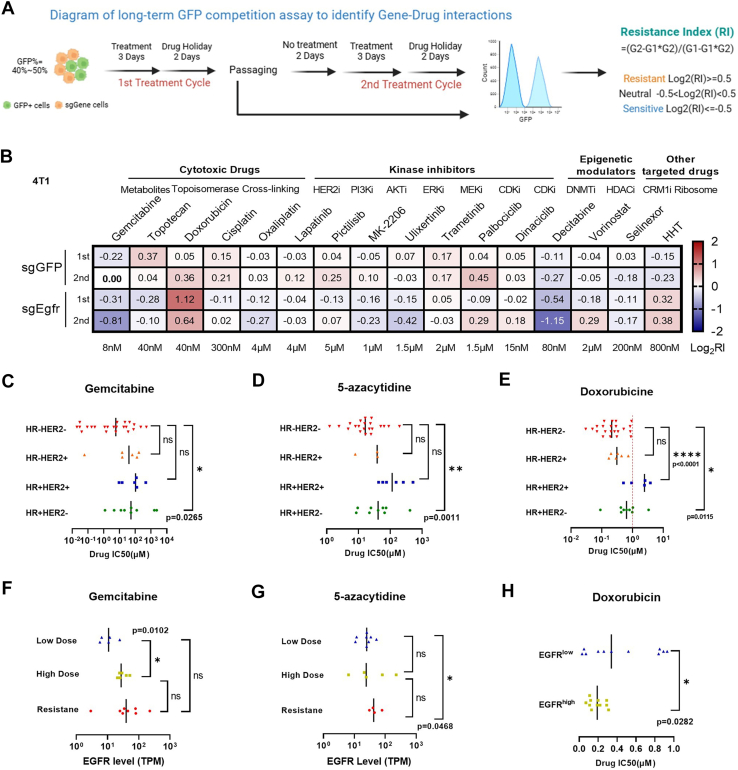
**Screening and identification of EGFR-interacting antitumor drugs.***A*, diagram of gene-drug interaction analysis. Initially, GFP-labeled control cells were mixed with GFP-negative EGFR-knockout cells. Subsequently, these mixed cells underwent two rounds of drug treatment. During this process, two GFP ratio detections were carried out, which were then used to calculate the resistance index (RI). *B*, gene–drug interaction pattern. *C*–*E*. The IC50 values of gemcitabine (*C*), 5-azacytidine (*D*), and doxorubicin (*E*) across different subtypes of breast cancer cell lines. *F* and *G*. The expression levels of EGFR in breast cancer cell lines exhibiting varying sensitivities to gemcitabine (*F*) and 5-azacytidine (*G*). *H*. The IC50 of doxorubicin in breast cancer cell lines with high and low EGFR expression. Multiple unpaired t-tests were used to test the significance.

To identify EGFR-interacting antitumor drugs, we designed a long-term GFP competition assay tailored for adherent cells to screen for gene-interacting drugs (Fig. 6*A*). This method was adapted from a previously reported approach (22). As the initial step of our research, we curated a panel of 33 antitumor drugs, all of which hold clinical or research significance in breast cancer therapy. We compared long-term (5-day) vs. short-term (2-day) gemcitabine (GEM) exposure on cell proliferation inhibition. The cytotoxic dose required for comparable suppression was about 10-fold lower for long-term (25 nM) than short-term (200 nM) treatment (Fig. S2*A*), highlighting the need to assess prolonged exposure in dose-response studies for accurate efficacy evaluation. Subsequently, we carried out dose gradient analysis to determine effective doses of these drugs for achieving long-term inhibitory actions (Fig. S2, *B–G*).

Ultimately, 16 agents were identified as a panel of breast cancer antitumor drugs, capable of exerting sustained inhibitory effects at concentrations below 10 μm. Following this, a mixed cell population of GFP + control cells and GFP- EGFR-knockout cells underwent two rounds of drug treatment. We calculated the resistance index by analyzing the change in GFP + proportion after drug treatment (Fig. 5*A*). Based on these data, we constructed an EGFR-Drug interaction pattern (Fig. 6*B*). Our findings unveiled an intriguing pattern: EGFR knockout led to increased sensitivity of tumor cells to gemcitabine (GEM) and decitabine (DAC), while simultaneously inducing tolerance to doxorubicin (DOX). These results imply a correlation between EGFR expression and drug sensitivity. Specifically, tumor cells with low-EGFR may exhibit sensitivity to GEM and DAC, whereas tumor cells with high-EGFR might be more responsive to DOX.

To validate the reliability of the EGFR-Drug interaction pattern, we analyzed the IC50 values of chemotherapeutic drugs in breast cancer cell lines from the GDSC database. Due to the lack of DAC data, we analyzed 5-azacytidine (5-AzaC), a drug with a similar mechanism of action. The results showed lower average IC50 values for GEM, 5-AzaC, and DOX in HR-HER2-cell lines compared to other subtypes (Fig. 6, *C*–*E*). Furthermore, based on GEM and 5-AzaC IC50 values, we classified HR-HER2-cell lines into high-dose responders, low-dose responders, and resistant cells (Fig. S3, *A* and *B*). We found that EGFR expression was significantly higher in resistant cells than in sensitive cells (Fig. 6, *F* and *G*). Since DOX IC50 values were very low (less than 1 μm) in all HR-HER2-cell lines, we divided them into EGFR-high and EGFR-low expressing groups (Fig. S3*C*). After that, we compared the IC50 values between these two groups. The outcome revealed that the cell lines with high-EGFR had lower IC50 values for DOX (Fig. 6*H*). Overall, these results provide strong evidence that the EGFR-Drug interaction pattern is valid and can be replicated in human HR-HER2-breast cancer cell lines.

In summary, these findings suggest that EGFR expression in triple-negative breast cancer (TNBC) serve as a potential predictive biomarker for drug response, aiding in chemotherapeutic drug selection and dosage determination.

### EGFR intervention boosts tumor cell sensitivity to chemo-drugs *via* ferroptosis induction

**Figure 7:**
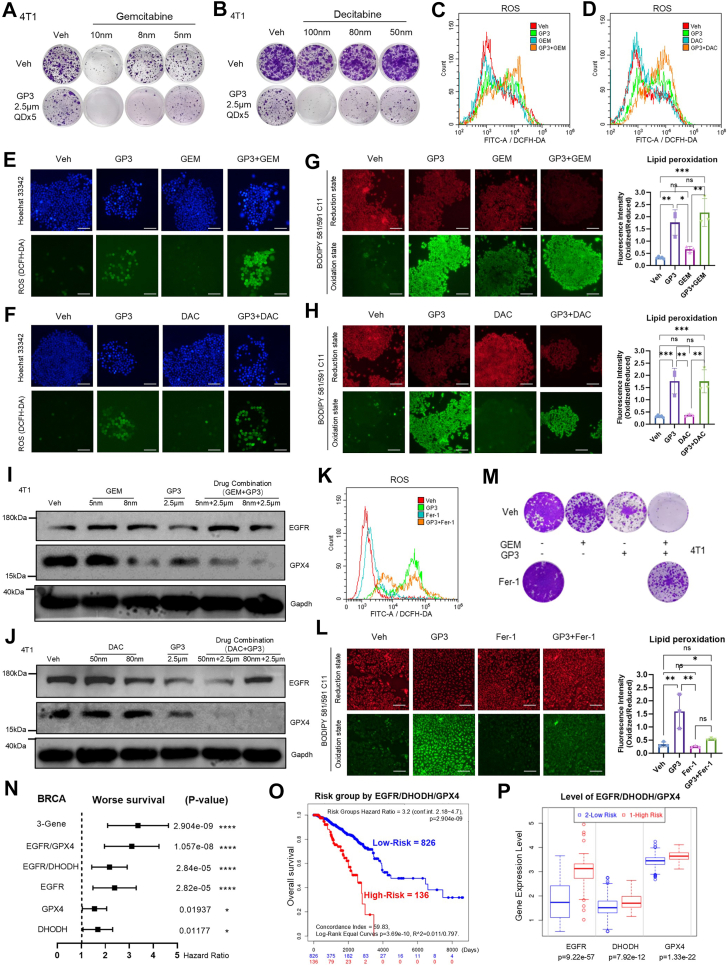
**The antitumor efficacy of combined EGFR intervention with GEM/DAC.***A* and *B*, effects of GP3+GEM (*A*) and GP3+DAC (*B*) on tumor cell clone formation. *C* and *D*. ROS levels in cells following GP3+GEM (*C*) and GP3+DAC (*D*)treatment analyzed by flow cytometry. GP3, 2.5 μm, QDx3. GEM, 5 nm. DAC, 80 nm. *E* and *F*, fluorescence images revealed ROS levels in cells following GP3+GEM (*E*) and GP3+DAC (*F*) treatment. GP3, 2.5 μm, QDx3. GEM, 5 nm. DAC, 80 nm. Bar is 200 μm. *G* and *H*. Lipid peroxidation levels in tumor cells after GP3+GEM (*G*) and GP3+DAC (*H*) treatment. GP3, 2.5 μm, QDx3. GEM, 5 nm. DAC, 80 nm. Bar is 200 μm. One-Way ANOVA tests were used to test the significance. *I* and *J*. Western blot analysis of apoptosis and ferroptosis markers in GP3+GEM (*I*) and GP3+DAC (*J*). *K*, ROS levels in cells following GP3+Fer-1 treatment analyzed by flow cytometry. GP3, 2.5 μm, QDx3. Fer-1, 5 μm. *L*. The effect of Fer-1 on GP3-induced LPO. GP3, 2.5 μm, QDx3. Fer-1, 5 μm. *M*. The effect of Fer-1 on GP3-induced ferroptosis susceptibility under GEM treatment. GEM, 10 nm. GP3, 2.5 μm, QDx5, Fer-1, 10 μm. *N*. Forest plot for the prognostic performance of DHODH, GPX4, EGFR, and their combinations. *p*-Value of the log-rank test were shown. A human BRCA dataset (TCGA-BRCA, n = 962) was chosen to survival analysis. The hazard ratio (HR), confidence interval, *p*-Value in forest plot were obtained from the SurvExpress program. *O* and *P*, survival curve of BRCA patients (*O*) and the expression level of DHODH, GPX4, EGFR (*P*) in low-risk and high-risk groups. The prognostic index (PI) was calculated by the joint expression level of the 3 genes and the Cox model to generate the risk groups. The optimization algorithm was applied in risk grouping by the SurvExpress program. One-way ANOVA with tukey’s multiple comparisons test were used to test the significance in (*G*), (*H*), (*L*).

Given that EGFR level is correlated with the sensitivity of tumor cells to chemotherapeutic agents, it is plausible that pharmacological intervention targeting EGFR could potentially enhance the cells' susceptibility to these drugs, thereby yielding sensitizing effects with reduced toxicity. Here, we independently evaluated the combined effects of the EGFR degrader GP3 and the EGFR inhibitor Erlotinib when used in conjunction with the GEM and DAC. Long-term drug treatment assays demonstrated that the combination of either the EGFR degrader or the EGFR inhibitor with GEM or DAC resulted in a stronger suppression of tumor cell clone formation (Figs. 7, *A* and *B* and S4, *A* and *B*).

To elucidate the molecular mechanisms underlying the enhanced effect between EGFR degrader/inhibitor and GEM/DAC, we first analyzed the correlation in drug response between GEM and DAC, as well as the potential pharmacological mechanisms of action of these two drugs. Upon analyzing the GDSC database, we discovered a significant positive correlation in the IC50 values of GEM and DAC in various types of tumor cell lines (Fig. S5, *A–D*). This finding strongly implies that GEM and DAC share a common mechanism of drug action. Furthermore, previous literature indicates that, unlike other chemotherapeutic drugs, both GEM and DAC exert their effects by competing with endogenous dCTP (23). Inhibition of DHODH, an enzyme that negatively regulates ferroptosis, restored gemcitabine sensitivity in pancreatic ductal adenocarcinoma (PDAC) tumor model (24). Notably, EGFR activity is closely associated with ferroptosis. Recent reports have demonstrated that the activation of EGFR signaling stabilizes DHODH, thereby conferring resistance to chemotherapy-induced ferroptosis (25). Given that EGFR degraders and inhibitors mainly affect lipid metabolism, we hypothesized that EGFR intervention augment cellular susceptibility to ferroptosis by boosting lipid metabolism, thereby effectively inducing ferroptosis when combined with GEM/DAC.

To test this hypothesis, we analyzed the reactive oxygen species (ROS) levels, a key initiator of lipid peroxidation, following treatment with either single drugs or drug combinations. Our findings indicate that the EGFR degrader GP3 facilitates ROS accumulation either when used alone or in combination with GEM/DAC (Fig. 7, *C*–*F*). Consistent with these observations, similar trends in lipid peroxidation levels were also detected (Fig. 7, *G* and *H*). Additionally, the EGFR inhibitor Erlotinib can also induce ROS accumulation and elevated lipid peroxidation levels (Fig. S4, *C* and *D*).

Next, we examined the protein levels of EGFR, phosphorylated γH2Ax for DNA double-strand breaks (DSBs), apoptosis markers and ferroptosis markers. Our findings revealed a notable downregulation of GPX4, a key anti-ferroptosis protein, following treatment with EGFR degrader/inhibitor and the drug combinations (Figs. 7, *I* and *J* and S4, *E*–*H*). These findings indicate that EGFR degraders or inhibitors, in combination with gemcitabine/decitabine, generate an enhanced inhibitory effect by promoting ferroptosis.

To further validate the EGFRi-induced susceptibility to ferroptosis, we employed the ferroptosis inhibitor Ferrostatin-1 (Fer-1), which significantly inhibited GP3-induced ROS across a concentration range of 0.1 to 5 μm (Fig. S4*I*). As expected, Fer-1 treatment effectively attenuated GP3-induced ROS elevation (Fig. 7*K*) and LPO (Fig. 7*L*), and consequent ferroptosis susceptibility under GEM treatment (Fig. 7*M*). These data collectively indicate that EGFRi induces a ferroptosis-sensitive state.

To consolidate our findings, we further validated the combination of GP3 and GEM in the human TNBC cell lines MDA-MB-231 and BT-549. Long-term inhibitory dose analysis showed that BT-549 exhibited greater tolerance to GEM, requiring a higher killing dose than 4T1 and MDA-MB-231 (Fig. S6, *A* and *B*). In both cell lines, GP3 can induce ROS production (Fig. S6, *C* and *D*). Moreover, the combination of GP3 and GEM demonstrated enhanced activity (Fig. S6, *E* and *F*), which was attenuated by Fer-1 (Fig. S6, *G* and *H*). Among the tested cell lines, BT-549 displayed the most obvious phenotype.

To better understand the clinical significance of EGFR and ferroptosis, as well as the potential of drug combinations for clinical application, we analyzed how the expression levels of EGFR, GPX4, and DHODH relate to the prognosis of breast cancer patients. The results indicated a statistically significant association between the expression levels of GPX4 and DHODH and patient prognosis. Of particular note, the joint expression of EGFR and GPX4 provided a more accurate prognostic prediction compared to EGFR expression (Fig. 7*N*). Moreover, the tri-gene (EGFR, GPX4, DHODH) joint expression emerged as the most powerful prognostic indicator (*p*-value of 2.904 × 10^−9^). Additionally, we observed an up-regulation of GPX4 and DHODH expression in the high-risk patient group (Fig. 7, *O* and *P*). In contrast, in other types of cancers, the associations and clinical correlations among EGFR, GPX4, and DHODH exhibit a notable lack of strength (Fig. S7). Together, these observations suggest that high EGFR and GPX4 expression are closely linked to poor outcomes in breast cancer. Targeting EGFR in combination with GEM/DAC, which can induce ferroptosis to achieve a chemosensitization effect, holds great promise as a therapeutic strategy for HR-HER2-EGFR^+^GPX4^+^ breast cancer patients.

### The EGFR degrader-GEM combo inhibits tumor lung colonization

**Figure 8:**
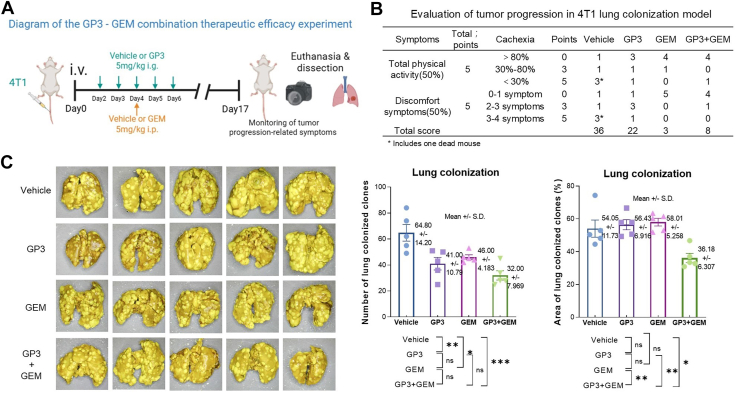
**Therapeutic efficacy of GP3+GEM combination in preventing lung colonization of tumor cells *in vivo*.***A*, schematic of tumor cell lung colonization model and treatment plan. *B*, phenotypic evaluation of disease progression in each group. *C*, images and quantification of tumor lung colonization. The number and area of colonized tumor clones were measured on both front and back surfaces of the lungs. Here, only front-view images are presented. One-way ANOVA with Tukey’s multiple comparisons test were used to test the significance.

To evaluate the *in vivo* efficacy of EGFR-targeting agents combined with chemotherapy drugs, we initially tested this regimen in a 4T1 lung colonization model (Fig. 8*A*). Given the risk of acquired resistance with kinase inhibitors, we employed the EGFR degrader (GP3). To enable the intracellular remodeling induced by EGFR degradation, GEM was administered 48 h after GP3 (Fig. 8*A*).

*In vivo* pharmacodynamic studies often use the Maximum Tolerated Dose (MTD) as a reference. However, we propose that MTD-based high-dose regimens, while effective, carry a dual risk: they can drive the emergence of acquired resistance and pose significant toxicity concerns. In this study, we aimed to investigate potential enhanced effects under conditions of limited single-agent efficacy. To this end, we deliberately selected a low dosage of GEM. Based on our long-term treatment assays (Fig. S2*A*), the inhibitory concentration for a 5-days regimen was 10- to 20-fold lower than that for a 2-days treatment. Accordingly, we used an *in vivo* GEM dose of 5 mg/kg—much lower than the typical range of 50 to 100 mg/kg reported by suppliers (Selleck, MCE). In the absence of established *in vivo* dosing guidelines for the EGFR degrader, we referred to the dosing regimens of conventional EGFR inhibitors. The recommended *in vivo* doses for inhibitors such as erlotinib and lapatinib typically range from 50 to 100 mg/kg, administered twice daily. For the EGFR degrader GP3, we selected a lower dose of 5 mg/kg, representing a 10- to 20-fold reduction from the reference range. This dose was administered once daily for five consecutive days, a regimen chosen in light of the compound's inherent instability and susceptibility to degradation.

To thoroughly assess the therapeutic efficacy of either single-drug treatment or drug combinations, we examined multiple parameters in mice. These included the malignant progression behavior of tumors, the number of lung colonized tumors, and the area occupied by these tumors. When examining the malignant progression of the disease in recipient mice, compared to the Vehicle group, GP3, GEM, and their combination all had shown therapeutic effects (Fig. 8*B* and Movies S3–S6). Among them, GEM and its combination have the most significant therapeutic impacts (Fig. 8*B* and Movies S3–S6). For the number of lung colonized tumors, all three treatment groups (GP3, GEM, and their combination) showed efficacy compared to the Vehicle group, and, the therapeutic effects were similar across these treatment groups (Fig. 8*C*). For the area of lung colonized tumors, only the drug combination group displayed a therapeutic effect when compared to the Vehicle group (Fig. 8*C*). By jointly analyzing the tumor number and area, we can gain insights into the relative size of individual tumors. Overall, these results indicate that the combination of the EGFR degrader GP3 and the chemotherapy drug GEM has an enhanced inhibitory effect in the tumor lung colonization model, providing a promising therapeutic strategy. Further optimization of the dosing frequency and dosage for both agents may enhance their interaction, thereby enhancing therapeutic outcomes.

## Discussion

This study reports the potential of EGFR levels as a biomarker for prognostic assessment and therapeutic efficacy prediction in triple-negative breast cancer (TNBC). Furthermore, we propose that EGFR degraders and inhibitors, in combination with gemcitabine/decitabine, exhibit enhanced effects, suggesting their potential as drug combinations. A rational approach to evaluating drug efficacy is crucial for determining the effectiveness of antitumor agents. However, tumor progression, particularly metastasis, is a highly complex process. The current reliance on cell proliferation inhibition as the primary metric for evaluating antitumor drugs imposes significant limitations and is a major contributor to the high failure rates observed in clinical trials. Consequently, establishing tumor metastasis models that closely mimic clinical scenarios is a key strategy for enhancing the accuracy of drug efficacy evaluations.

In this study, we simulated clinical scenarios by employing an orthotopic breast cancer allograft model and performing surgical resection of the primary tumors to facilitate the observation of tumor recurrence and progression. Without surgical intervention, mice typically reached the experimental endpoint within a relatively short period, with lung metastases serving as the primary hallmark of malignant tumor progression. Following surgical resection, we observed a more diverse pattern of tumor dissemination and metastasis. Notably, while mice in the sgEgfr group exhibited reduced lung metastases, there was an increased incidence of invasion into other organs, including the kidneys, liver, and thoracic vertebrae. We propose two potential explanations for the systemic metastasis observed upon EGFR knockout. First, the effects induced by EGFR knockout itself, such as alterations in the composition of adhesion proteins in tumor cells, may lead to changes in organotropism. Second, the impact of the surgical removal of the primary tumor could also play a role. Assuming that the duration of tumor cell dormancy and subsequent regrowth varies at different sites, with pulmonary tumor cells having a shorter dormancy period compared to those in other organs, the removal of the primary tumor might directly reduce lung metastases. This, in turn, prolongs the survival of mice, allowing tumor cells in other organs to form detectable tumors.

In this study, we employed a lung colonization model established *via* tail-vein injection to conduct a preliminary evaluation of the lung colonization capacity of tumor cells, as well as the therapeutic efficacy of the drug combination *in vivo*. Despite the relatively distinct phenotypic outcomes observed in the relevant experiments, we believe that the conclusions still necessitate validation using more physiologically relevant models. We hold the view that the lung colonization model based on tail-vein injection is highly artificial and lacks reliability. Although it is commonly regarded as a lung metastasis model in most studies, in reality, this model only simulates the colonization of circulating tumor cells in the lungs, a single step in tumor metastasis. Moreover, during tail-vein injection, tumor cells are directly injected into the vein. The blood circulation initially transports these cells to the lungs, where they become trapped and grow in the pulmonary capillaries. This process differs significantly from genuine tumor metastasis to the lungs, which involves a series of complex steps, including tumor cell invasion into surrounding tissues, intravasation into blood or lymphatic vessels, survival in the circulation, transform into circulating tumor cells, and eventual extravasation and colonization at distant sites.

Adopting appropriate drug dosages, prolonged treatment durations, and clinically meaningful correlations and discussions of drug sensitivity results are crucial elements for enhancing the accuracy of preclinical drug efficacy evaluations. In this study, we assessed drug inhibitory effects using prolonged tumor cell cultures spanning over five days, which contrasts sharply with short-term experimental approaches that evaluate cell numbers based on cell viability. For our long-term efficacy assessments, we employed lower drug doses as inhibitory concentrations, thereby bolstering the rationality of our experimental design and the precision of our efficacy evaluations (14). This approach effectively eliminates the confounding effects of responses induced by excessive drug concentrations.

Targeted protein degradation (TPD) represents a promising new strategy for drug development, yet its mode of action remains an area requiring in-depth exploration (26). In this study, we unexpectedly discovered that both EGFR degraders and EGFR inhibitors, at their effective inhibitory doses, are capable of inducing transcriptomic alterations in lipid metabolism signaling pathways. This suggests that the effects of EGFR degraders might resemble those of EGFR inhibitors. Despite the initial expectation that EGFR degraders, due to their design, would produce distinct effects from EGFR inhibitors by degrading EGFR protein (*i.e.*, protein degradation rather than merely inhibiting kinase activity), our analysis revealed that high-dose EGFR degraders, although unable to degrade EGFR protein due to the hook effect, still exerted significant inhibitory effects. These findings underscore the necessity of rigorous experimental evaluations, conducted under well-defined dosage conditions, to ascertain whether targeted protein degradation strategies exert their effects through protein degradation. For example, kinase inhibitors have long been thought to function primarily by blocking kinase activity and downstream signaling pathways. Yet a recent study has shown that nearly one in ten (160/1570) kinase inhibitors actually exert their effects through the degradation of target proteins (27). The unexpected phenomena and the underlying molecular mechanisms warrant further investigation in our future work. In summary, based on the findings of this study, we propose that EGFR kinase inhibitors and the EGFR PROTAC are not vastly different in their pharmacological outcomes at low doses, where both likely promote lipid metabolism/lipid peroxidation. However, considering the multi-faceted advantages of PROTACs over traditional inhibitors—such as their catalytic, substoichiometric mode of action leading to complete target elimination, and their potential to overcome resistance mutations—we recommend the EGFR PROTAC as the preferred strategy.

Ferroptosis represents a crucial mode of cell death in tumor cells; however, ferroptosis inducers face challenges in drug development due to various factors (28). As targeted agents, EGFR inhibitors or degraders are primarily expected to inhibit proliferation rather than induce direct cell death. Consistent with this, even complete EGFR knockout does not markedly affect cell survival in the DepMap database (Fig. 3*G*). Despite their lack of anti-proliferative activity, EGFR inhibitors or degraders still exert important biological effects. As our transcriptomic data show, they significantly upregulate lipid metabolism pathways (Fig. 5). Similarly, they can induce ROS and lipid peroxidation (LPO) (Fig. 7). Thus, at our experimental doses, single-agent EGFR inhibitors/degraders induce ROS and LPO without causing proliferation arrest or cell death. This indicates that ROS and LPO are necessary but insufficient conditions for ferroptosis. In contrast to single agents, the drug combination markedly downregulated GPX4. This specific suppression of a key ferroptosis defense is our primary evidence that the combination's mechanism is dependent on ferroptosis induction itself, not merely on elevated ROS. This conclusion aligns with recent findings, which identify the knockout or inhibition of GPX4 as a critical event that sensitizes cells to the CDK4/6 inhibitor Palbociclib and the ER degrader Giredestrant (29). *In vivo* experiments confirmed that GPX4 deficiency confers marked sensitivity to Palbociclib. The intrinsic undruggability of GPX4, however, presents a major barrier to clinical application. Notably, GPX4 downregulation was observed only under the drug combination (with similar results in both the GEM and DAC groups), whereas EGFR degradation remained inconspicuous. One possible explanation is that GEM-induced cell cycle arrest slows protein synthesis, thereby reducing the availability of regulatory proteins required for EGFR degradation. This may alter the stoichiometry of the degradation complex, trigger a hook effect, destabilize the ternary complex, and consequently impair GP3-induced EGFR degradation. As previously discussed, the failure of GP3 to induce EGFR protein degradation—whether due to the hook effect or other causes—does not necessarily indicate that EGFR function and activity remain intact. GP3 may operate through alternative pathways, such as modulating the transcription of genes involved in lipid metabolism or altering EGFR phosphorylation activity. In summary, we propose that ferroptosis is the core mechanism of the drug combination. The role of the EGFR-targeting agents is to prime tumor cells into a pro-oxidant state (high ROS/LPO), creating a necessary precondition. The combination then exploits this precondition by inhibiting GPX4, ultimately leading to ferroptotic cell death.

In this study, leveraging a gene-drug interaction screening system, we identified gemcitabine/decitabine as interacting drugs of EGFR. Our findings demonstrate that targeting EGFR with degraders or inhibitors sensitizes cancer cells to gemcitabine/decitabine, *via* the induction of ferroptosis. Gemcitabine is already a first-line chemotherapy agent for breast cancer, and EGFR inhibitors are approved clinical drugs for multiple tumor types. Consequently, the combination of EGFR inhibitors with gemcitabine presents a highly feasible drug-combination strategy with substantial potential for clinical translation.

## Experimental procedures

### Cell lines

Mouse breast cancer cell lines 4T1 and EO771 were obtained from the Cell Bank of the Chinese Academy of Sciences and Hunan Fenghui Biotechnology Co, Ltd, respectively. 4T1 cells were cultured in RPMI 1640 medium, while EO771 cells were maintained in DMEM. Human breast cancer cell lines MDA-MB-231 and BT-549 were from our laboratory stock and cultured in DMEM and RPMI 1640 medium, respectively. All media were supplemented with 10% fetal bovine serum (FBS), 100 U/ml penicillin, and 100 μg/ml streptomycin. Cell line authenticity was verified by RNA sequencing or STR. Additionally, routine PCR-based screening for *mycoplasma* contamination is conducted every two weeks to ensure cell line integrity.

### Establishment of stable cell lines

sgRNAs targeting either GFP or murine EGFR were designed using the Benchling website, synthesized, and subsequently cloned into the lentiCRISPR v2 vector. The sequences for the sgRNAs are as follows:

sgGFP-Top: 5′-CACCGCATGCCGAGAGTGATCCCGG-3′;

sgGFP-Bottom: 5′-AAACCCGGGATCACTCTCGGCATGC-3′;

sgEgfr-Top: 5′-CACCGGTCAGCAACACCAGCAGTG-3′;

sgEgfr-Bottom: 5′-AAACCACTGCTGGTGTTGCTGACC-3′.

Lentiviral vectors, along with packaging plasmids, were transfected into 293T cells using the calcium phosphate precipitation method. Viral supernatants were harvested 48 h post-transfection. 4T1 cells were infected with viruses carrying either a control sgRNA-targeting GFP (sgGFP) or an sgRNA targeting EGFR (sgEgfr). Stable cell lines were established through puromycin selection.

### Chemicals

Chemotherapy drugs: 5-Fluorouracil (5-FU, S1209), Gemcitabine (GEM, S1714), Cytarabine (Ara-C, S1648), Methotrexate (MTX, S1210), Paclitaxel (PTX, S1150), Vincristine (VCR, S1241), Topotecan (TPT, S1231), Doxorubicin (DOX, S1208), Cisplatin (CDDP, S1166), Oxaliplatin (OXA, S1224), Thioguanine (6-TG, S1774) were purchased from Selleck, USA.

Kinase inhibitors: Erlotinib (Erlo, S1023), Lapatinib (Lapa, S1028), Pictilisib (Pic, S1065), MK-2206 (S1078), Rapamycin (Rapa, S1039), Caffeic Acid Phenethyl Ester (CAPE, S7414), Sorafenib (Sora, S1040), Trametinib (Trame, S2673), Ulixertinib (Ulixer, S7854) Palbociclib (Palbo, S1116) and Dinaciclib (Dina, S2768), were purchased from Selleck.

Epigenetic modulators: Decitabine (DAC, S1200), Vorinostat (SAHA, S1047), Pemrametostat (Pemr, S8664), Tazemetostat (Taze, S7128) and Curcumin (S1848) were purchased from Selleck. Molibresib (Moli, HY-13032) and Birabresib (Bira, HY-15743) were purchased from MCE, USA.

Other targeted drugs: Venetoclax (Vene, S8048), Navitoclax (Navi, S1001), AZD2281 (S1060), Selinexor (Seli, S7252) and Homoharringtonine (HHT, S9015) were purchased from Selleck, USA.

The ferroptosis inhibitor Ferrostatin-1 (Fer-1, HY-100579) and Gefitinib-based PROTAC 3 (GP3, HY-123921) were purchased from MCE. Water-soluble gemcitabine hydrochloride (S1149) for *in vivo* experiments was purchased from Selleck, USA.

Most chemicals were dissolved in dimethyl sulfoxide (DMSO, V900090, Merck) and to concentrations of 10 mM and aliquoted and stored at −20 °C. CDDP and OXA were dissolved in Dimethylformamide (DMF, D4551, Merck) and stored at −80 °C. GP3 was stored at −80 °C. Erlotinib, OXA and HHT were dissolved to concentrations of 5 mM. GEM, PTX, VCR and Dinaciclib were dissolved to concentrations of 100 μm. Working concentrations for all chemicals were determined by inhibitory dose tests.

### 4T1-OtR model and 4T1 lung colonization model

The 4T1-OtR model was employed to simulate orthotopic tumor growth and tumor dissemination following surgical resection. BALB/c mice aged 6 to 8 weeks were anesthetized with 220 to 250 μl of 2.5% Avertin per mouse. A suspension of 1 × 10^5^ cells in 50 μl of 1 × DPBS was injected into the left fourth mammary fat pad of each mouse. Orthotopic tumor dimensions were measured starting on day 5 post-injection, and tumor volumes were calculated using the formula: volume = (length × width^2^) × one-third. Once tumors reached 400 to 600 mm^3^, mice were anesthetized again, and orthotopic tumors were surgically excised, followed by suture closure of the surgical site. Mice were monitored daily thereafter. Upon presentation of disease progression-related symptoms, mice were euthanized and dissected to assess sites of tumor dissemination and metastasis.

The 4T1 lung colonization model was utilized to evaluate the impact of genetic manipulations or pharmacological interventions on the process of tumor cell colonization and growth in the lungs. A suspension of 1 × 10^5^ cells in 200 μl of 1 × DPBS was administered *via* tail vein injection to 6–8-week-old BALB/c mice. Mice were closely monitored for signs of tumor progression, including assessments of locomotor activity and overt signs of distress, such as vertical hair, hunched posture, tremors, and prolonged eye closure. The experimental endpoint was defined as the occurrence of distinct tumor progression-related symptoms in two or more mice within a cohort. Video recordings and detailed documentation of locomotor impairment and distress symptoms were conducted prior to euthanasia. Subsequently, mice were euthanized and dissected to examine pulmonary tumor burden and invasion.

Lung tissues colonized by tumors were stained using Bouin's fixative, and images of both the front and back surfaces of the lungs were captured under a stereomicroscope. Subsequently, the lung images were analyzed using ImageJ software to quantify the number and area of pulmonary tumor colonies.

BALB/c mice were purchased from Laboratory Animal Research Center in Jiangsu University. This study used an experimental female breast cancer model, employing exclusively female mice to maintain gender-specific relevance.

All mice were housed in a specific pathogen-free environment at Laboratory Animal Research Center in Jiangsu University and treated in strict accordance with protocols, which were approved by the Animal Care and Use Committee of Laboratory Animal Research Center, Jiangsu University.

### Cell proliferation dependency analysis

The GFP competition assay was adapted to analyze EGFR-dependent cell proliferation. EGFR-knockout (EGFR-KO) cells lacking GFP expression were mixed with GFP-positive cells expressing endogenous EGFR. The mixed cell populations were cultured and propagated under diverse conditions, including standard growth media, pharmacological treatments, or serum deprivation. GFP ratios were dynamically monitored across cell passages to assess the impact of EGFR status on proliferation under these experimental conditions.

All flow cytometry analyses were conducted using the CytoFlex instrument (Beckman) and the data were thoroughly analyzed utilizing CytExpert software (Beckman).

### Gene-drug interaction analysis

A panel of breast cancer antitumor agents suitable for GFP competition assays was established. 33 antitumor drugs relevant to breast cancer treatment were evaluated for their long-term inhibitory effects on tumor cells using dose-response analyses (Fig. S2, *B–E*). For cytotoxic agents, the maximum test dose was set at 1 μm, while for other drugs, it was set at 10 μm. Drugs that failed to support tumor cell passaging and proliferation following treatment—including paclitaxel (PTX), vincristine (VCR), and dinaciclib—were excluded. Ultimately, 16 drugs were selected to constitute the breast cancer antitumor drug panel.

The GFP competition assay was modified as follows for Gene-Drug interaction analysis. Mixed cell populations (4000 cells per well) were seeded into 12-well plates, with the vehicle control group receiving one-third of this cell density. Drug treatment was initiated two days post-seeding. After three days of drug exposure, the media was replaced. Tumor cells were allowed to grow for an additional two days before being harvested for passaging and flow cytometry analysis of GFP ratios. The vehicle control group was passaged at a 5:1 ratio, while the passaging ratio for drug-treated groups was adjusted based on cell counts per well. Following two days of growth post-passaging, a second round of drug treatment and GFP ratio analysis was conducted.

During cell harvesting and passaging, a small aliquot of cells was collected for GFP ratio analysis. After staining with propidium iodide (PI) for 10 min, flow cytometry was performed to analyze the GFP ratio in viable (PI-negative) cells. Following two rounds of drug treatment, the GFP ratios of vehicle control and drug-treated groups were used to calculate the resistance index (RI), which was employed to evaluate the impact of genetic manipulations on therapeutic response of cells to tested drugs. RI = (G1-G1∗G2)/(G2-G1∗G2). G1 = 100%-NonG1. NonG1, GFP% in vehicle control. G2 = 100%-NonG2. NonG2, GFP% in drug treated.

### Long-term drug treatment assay

The long-term drug treatment assay was conducted in 24-well plates, with cells seeded at a density of 2000 cells per well. Drug treatment was initiated 48 h post-seeding using 500 μl of drug-containing media per well. After 3 days of incubation, 300 μl of fresh media was added to each well. Typically, vehicle control wells reached confluence 5 to 6 days post-treatment, at which point standard crystal violet staining was performed. In the clone formation assay, cells were seeded at 100 cells per well and Crystal violet staining was performed at 9 to 10 days post-treatment.

For combination drug treatments, 250 μl of 2 × drug-containing media was added to the respective wells. For sequential GP3 treatment, 100 μl of 5 × drug-containing media was supplemented daily from day 2 to day 4. In all experiments, appropriate volumes of media were added to vehicle control, single-drug, or single-dose treatment groups to maintain consistency.

### ROS level analysis

To detect intracellular reactive oxygen species (ROS) levels in 4T1 cells following drug treatment, cells were seeded into a 24-well plate at a density of 2000 cells per well and incubated for 48 h to allow attachment and growth. After treatment with individual drugs or drug combinations for 72 h, ROS was measured using a commercial detection kit (Beyotime, S0033S) with modifications to the manufacturer’s protocol: the DCFH-DA probe was diluted 1:5000 in PBS, and 100 μl of the diluted solution was added to each well for incubation at 37 °C in the dark for 30 min. Cells were then washed twice with PBS, followed by nuclear counterstaining with Hoechst 33,342 (1 μg/ml) added at 100 μl per well and incubated at 37 °C in the dark for 10 min. After two additional PBS washes, 100 μl of PBS was added to each well, and ROS fluorescence was visualized using a fluorescence microscope (Nikon, Ti2-U). Fluorescence photographs within the same fluorescence channel were captured and examined with the same exposure settings.

Flow cytometry analysis was conducted employing experimental procedures similar to those described above, along with identical experimental parameters. The only modifications made were the incorporation of a cell digestion step and the elimination of the Hoechst 33,342 staining step. All flow cytometry analyses were conducted using the CytoFlex instrument (Beckman) and the data were thoroughly analyzed utilizing CytExpert software (Beckman).

### Lipid peroxidation analysis

To detect lipid peroxidation (LPO) levels in 4T1 cells following drug treatment, cells were seeded into a 24-well plate at a density of 2000 cells per well and incubated for 48 h to allow attachment and growth. After treatment with individual drugs or drug combinations for 72 h, Lipid peroxidation was measured using a commercial detection kit (Beyotime, S0043S) with modifications to the manufacturer’s protocol: the BODIPY 581/591 C11 (2 mM) probe was diluted 1:400 in PBS, and 250 μl of the diluted solution was added to each well for incubation at 37 °C in the dark for 60 min. Cells were then washed twice with PBS. Following the washes, 500 μl of PBS was added to each well, and red fluorescence (indicative of the reduced state) and green fluorescence (indicative of the oxidized state) were visualized using a fluorescence microscope (Nikon, Ti2-U). Fluorescence photographs within the same fluorescence channel were captured and examined with the same exposure settings.

### Transcriptome bulk-sequencing

Cells were preserved in Trizol at −80 °C for RNA extraction. Two biological replicates were employed for each sample. Total RNA was extracted using Trizol reagent kit (15596018, Invitrogen) according to the manufacturer’s protocol. The total RNA samples were transported on dry ice to Gene Denovo Biotechnology Company for library construction and bulk-sequencing (Illumina Novaseq6000). Differentially expressed genes analysis was performed by DESeq2 software between two different groups. The genes with the parameter of false discovery rate (FDR) below 0.05 and absolute fold change≥2 or ≥1.5 were considered differentially expressed genes (DEGs). Pathway enrichment analysis enrichment of DEGs were performed with Metascape (https://metascape.org).

### Protein extraction and Western blot analysis

4T1 cells cultured in 6-well plate were lysed using RIPA buffer on ice. After the addition of loading buffer, the samples were heated at 95 °C for 10 min and stored at −20 °C for later use. For Western Blot analysis, SDS-PAGE gels were employed to separate proteins, followed by standard blotting and detection procedures. Rabbit monoclonal anti-EGFR (SDT-R110, STARTER, 1:1000), rabbit monoclonal anti-Phospho-EGFR(Tyr1068) (3777T, CST, 1:1000), rabbit monoclonal anti-Phospho-AKT(Ser473) (4060T, CST, 1:2000), rabbit polyclonal AKT (A7270, Abclonal, 1:1000), rabbit monoclonal anti-Phospho-ERK1/2(Thr202/Tyr204) (4370T, CST, 1:2000), rabbit polyclonal ERK1/2 (BS1112, Bioworld, 1:500), rabbit monoclonal anti-Phospho-H2AX(S139) (AP0687, Abclonal, 1:1000), rabbit monoclonal anti-Caspase-3(active + pro) (A19654, Abclonal, 1:1000), rabbit monoclonal anti-CDKN1A/p21 (A19094, Abclonal, 1:1000), and rabbit monoclonal anti-GPX4 (ab125066, Abcam, 1:1000) were employed for the study. Rabbit monoclonal anti-Tubulin (AC012, Abclonal, 1:5000) and mouse monoclonal anti-GAPDH (AC002, Abclonal, 1:10000) were used as internal control. The specificity of each antibody used has been validated by the company through methods such as Western blot. The uncropped, original Western Blot images were included in the (Fig. S8).

### Prognostic analysis

Survival analysis was conducted utilizing the online SurvExpress tool (30). The prognostic index was derived by the expression value and employed a Cox model to stratify patients into distinct risk groups. The risk grouping was further optimized using an integrated algorithm within the SurvExpress program.

### Statistical analysis

The statistical methods employed were tailored to the specific type of experiment, including unpaired t-tests, One-Way ANOVA tests, and log-rank (Mantel–Cox) tests, as detailed in the respective figure legends. Data were presented as mean ± SD. Statistical significance was determined by *p* values less than 0.05. Significance levels were denoted as follows: ∗*p* < 0.05, ∗∗*p* < 0.01, ∗∗∗*p* < 0.001, and ∗∗∗∗*p* < 0.0001. Statistical analyses were conducted using GraphPad Prism 9.0 software (GraphPad Software).

## Ethics approval

The Animal Care and Use Committee of Laboratory Animal Research Center at Jiangsu University approved all animal procedures (Approval No.: UJS-IACUC-2024092401).

## Data availability

All raw data generated or analyzed throughout this study are accessible upon reasonable request from the corresponding author. RNA-Seq data pertaining to this investigation have been deposited in the NCBI Sequence Read Archive (SRA) under the following accession codes: PRJNA1267460. Any cell lines or strains generated in this study can be acquired through the Materials Transfer Agreement (MTA). Further information and requests for resources and reagents should be directed to Xiaoxi Li (lixiaoxi@ujs.edu.cn).

## Supporting information

This article contains supporting information.

## Conflict of interest

The authors declare that they have no conflicts of interest with the contents of this article.
