# Supplementary material for: Low-dose EGFR inhibition unlocks ferroptosis susceptibility to sensitize chemotherapy in EGFR-high triple-negative breast cancer
Source: J Biol Chem. 2026 May 25;302(7):113185. doi: 10.1016/j.jbc.2026.113185 (PMC13284506; doi:10.1016/j.jbc.2026.113185)
Supplement: Supplementary Material [file mmc1.pdf]

Supporting Information

Low-dose EGFR inhibition unlocks ferroptosis susceptibility to sensitize chemotherapy in EGFR-high triple-negative breast cancer

Corresponding authors: lixiaoxi@ujs.edu.cn (Xiaoxi Li)

List of Supplementary Materials

Supplementary Figure 1–7

Fig. S1. Photographs of primary tumors and metastatic lesions.

Fig. S2. Determination of the inhibitory doses for antitumor drugs.

Fig. S3. The IC50 values of antitumor drugs and their response types.

Fig. S4. The synergistic effects between the EGFR inhibitor and GEM/DAC.

Fig. S5. Correlation of responses to Gemcitabine and 5-Azacytidine in multiple tumor cell lines.

Fig. S6. The synergistic effects of GP3 and GEM in the human TNBR cell lines.

Fig. S7. Clinical relevance of DHODH, GPX4, EGFR expression.

Fig. S8. Uncropped original Western Blot images.

Supplementary Movie 1–6

Movie S1. Locomotor activity in recipient mice with 4T1-sgGFP.

Movie S2. Locomotor activity in recipient mice with 4T1-sgEgfr.

Movie S3. Locomotor activity in the vehicle group of recipient mice with 4T1.

Movie S4. Locomotor activity in the GP3 group of recipient mice with 4T1.

Movie S5. Locomotor activity in the GEM group of recipient mice with 4T1.

Movie S6. Locomotor activity in the GP3+GEM group of recipient mice with 4T1.

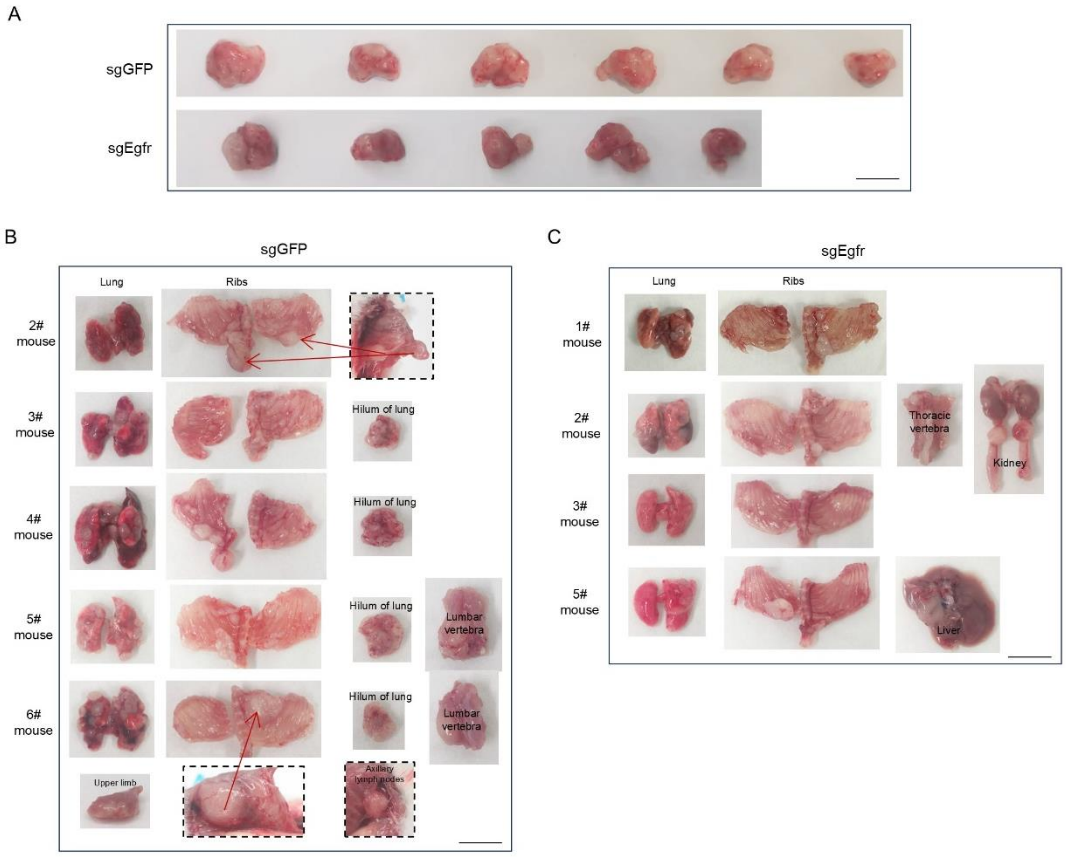

Fig. S1. Photographs of primary tumors and metastatic lesions. A. Excised primary tumors following surgical resection. n(sgGFP)=6, n(sgEgfr)=5. The tumors were of comparable size, with minimal intra-group variability. B–C. Visible metastatic tumors during dissection of sgGFP mice (B) and sgEgfr mice (C). n(sgGFP)=5, n(sgEgfr)=4. Bar lengths indicate 1 cm. The images within dashed lines lack a calibrated scale.

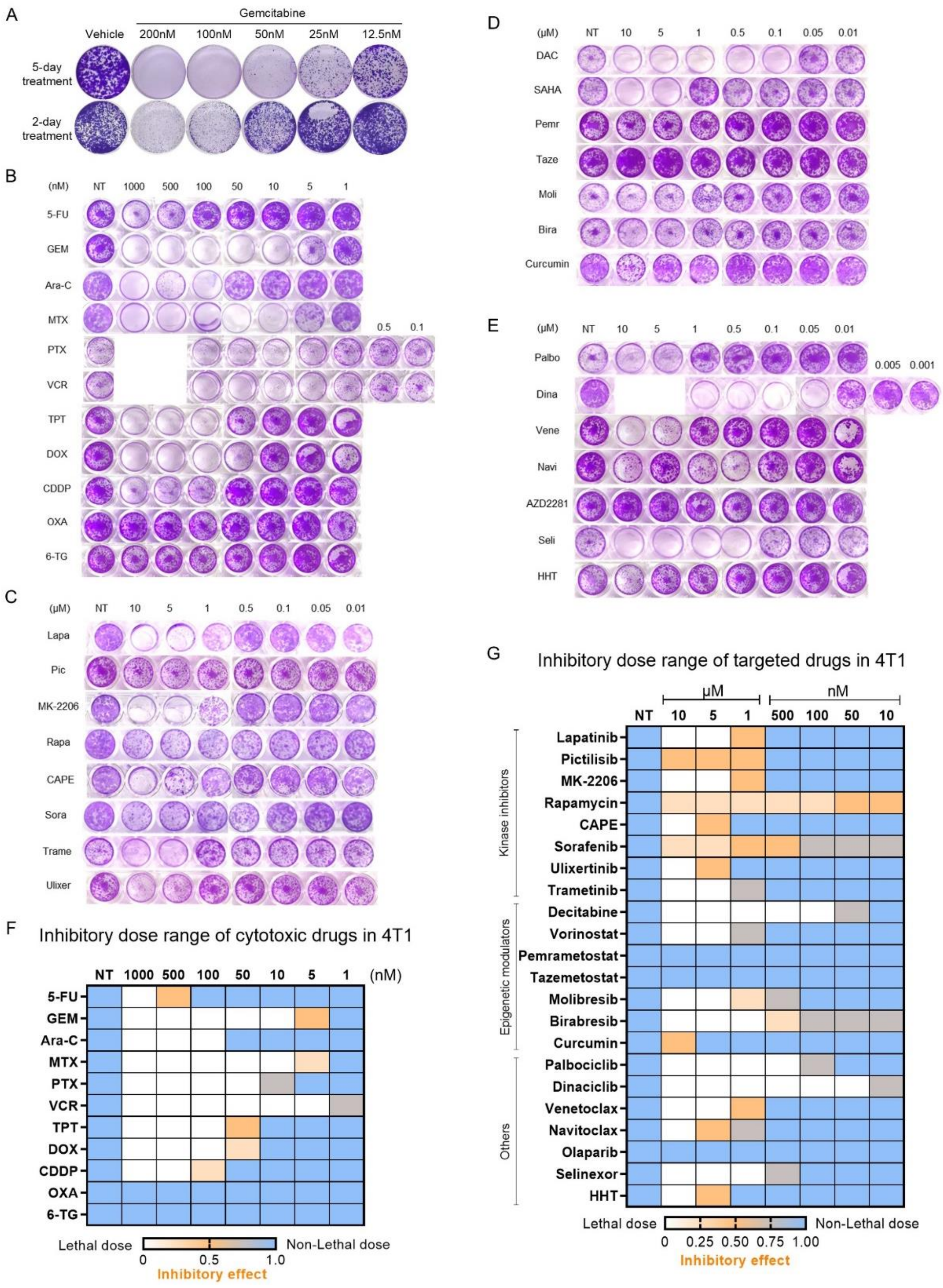

Fig. S2. Determination of the inhibitory doses for antitumor drugs. A. Comparison of the inhibitory effects of gemcitabine treatment duration (5 Days vs. 2 Days) on cell proliferation. Cells were seeded at 2×10^3 cells or 6×10^3 per well in 24-well plates for the 5-day or 2-day experiments. B-D. Long-term inhibitory dose of chemotherapeutic drugs (B), kinase-targeted inhibitors (C), epigenetic modulators (D), and other targeted drugs (E) against 4T1 cells. F-G. Schematic diagram illustrating the effective inhibitory doses of chemotherapeutic drugs (F) and targeted drugs (G).

A

HR-HER2- cell lines	GEM IC50 (μM)	Drug Response & Drug Dose
HCC1187	505.14	Resistance (IC50>10μM)
HCC1937	225.29	
HCC1395	84.94	
HCC2157	43.22	
HCC70	35.55	
BT-549	27.00	
HCC1143	22.15	
BT-20	18.72	
Hs-578-T	7.11	High Dose (IC50:1~10μM)
CAL-120	6.49	
HDQ-P1	5.89	
CAL-85-1	4.40	
MDA-MB-436	1.76	
MDA-MB-231	1.43	
MDA-MB-157	0.27	Low Dose (IC50<1μM)
CAL-148	0.09	
HCC1599	0.04	
HCC38	0.03	
CAL-51	0.02	

B

HR-HER2- cell lines	5-AzaC IC50 (μM)	Drug Response & Drug Dose
HCC1143	192.98	Resistance (IC50>50μM)
HCC1937	89.13	
BT-549	63.82	
CAL-120	50.03	
CAL-85-1	26.99	High Dose (IC50:20~30μM)
BT-20	25.65	
CAL-148	24.66	
HDQ-P1	23.61	
HCC70	21.92	
HCC1395	16.67	Low Dose (IC50<20μM)
MDA-MB-231	16.31	
HCC38	15.59	
MDA-MB-436	14.93	
MDA-MB-157	11.54	
CAL-51	11.31	
Hs-578-T	10.22	
HCC1187	4.74	

C

HR-HER2- cell lines	EGFR (TPM)	DOX IC50 (μM)	EGFR level
MDA-MB-468	580.01	0.07	EGFR^{high} cell lines
BT-20	224.80	0.19	
HCC70	80.11	0.29	
HCC1143	78.77	0.22	
MDA-MB-231	51.95	0.11	
BT-549	44.93	0.11	
CAL-120	39.79	0.31	
HCC1187	34.21	0.12	
HCC1937	30.72	0.29	
Hs-578-T	29.02	0.20	
MDA-MB-436	25.32	0.13	
HCC1395	25.04	0.84	EGFR^{low} cell lines
HCC38	24.58	0.20	
CAL-85-1	24.24	0.26	
HDQ-P1	20.68	0.85	
CAL-51	11.80	0.03	
MDA-MB-157	10.76	0.34	
HCC1599	5.73	0.07	
HCC2157	2.92	0.93	
DU-4475	0.16	0.05	
MDA-MB-453	0.11	0.52	
MFM-223	0.08	0.89	

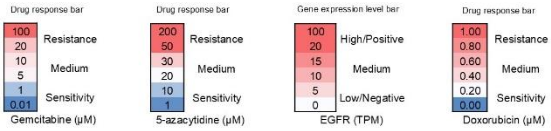

Fig. S3. The IC50 values of antitumor drugs and their response types. A-B. The IC50 values of GEM (A) and 5-AzaC (B) in HR-HER2- breast cancer cell lines and their response types. 1 μM is the maximum effective dose of GEM. 20 μM is the maximum effective dose of 5-AzaC. C. The IC50 values of DOX and EGFR expression levels in HR-HER2- breast cancer cell lines. Due to the presence of aberrant data from individual cells, which may suggest the involvement of alternative underlying mechanisms, the relevant data were manually excluded from the analysis.

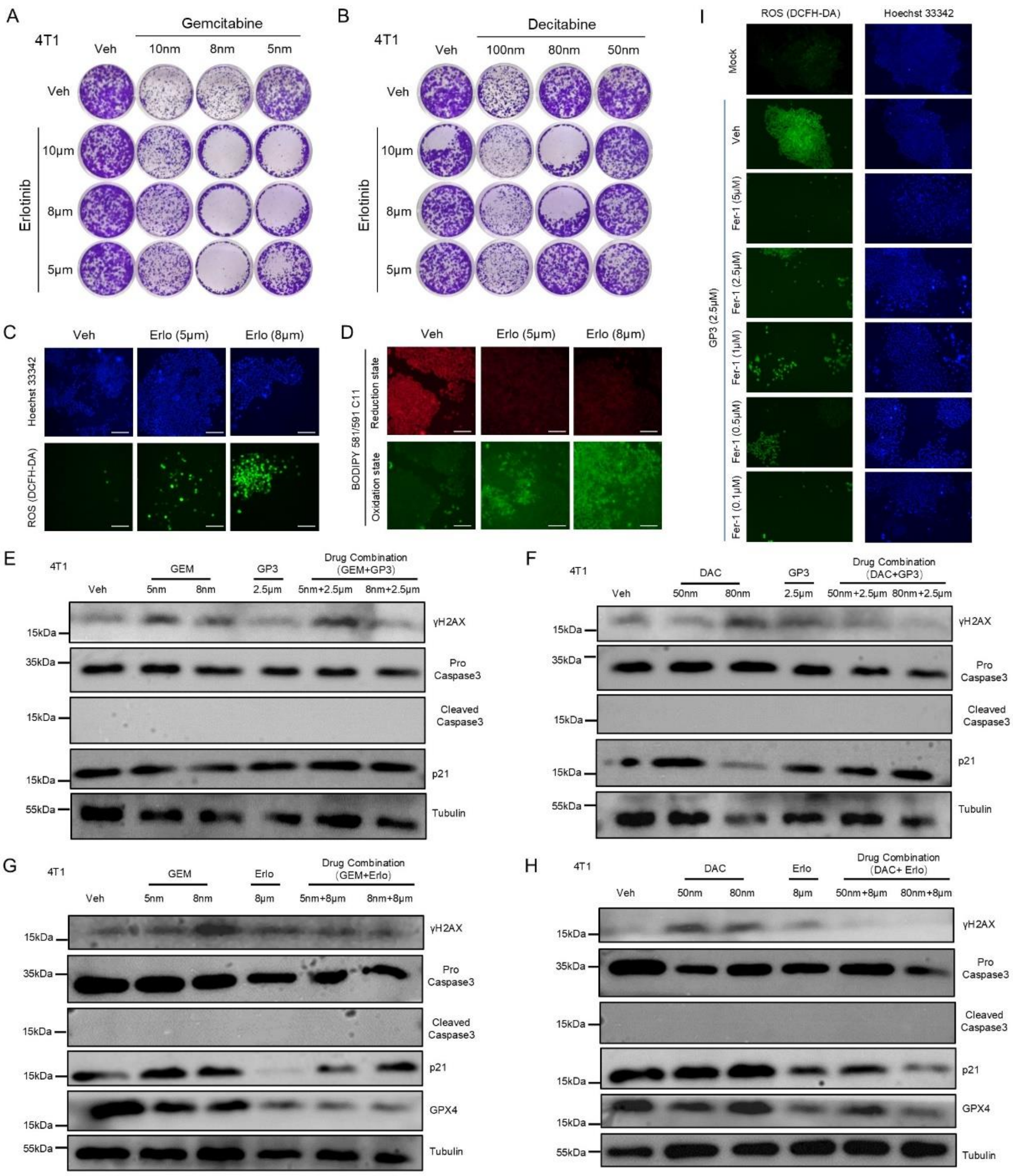

Fig. S4. The synergistic effects between the EGFR inhibitor and GEM/DAC. A-B. Effects of Erlotinib + GEM (A) and Erlotinib + DAC (B) on tumor cell clone formation. C-D. ROS levels (C) and lipid peroxidation levels (D) in tumor cells after Erlotinib treatment. Erlo, Erlotinib. Bar is 200μm. E-F. Western blot analysis of apoptosis markers in GP3+GEM (E) and GP3+DAC (F). G-H. Western blot analysis of apoptosis and ferroptosis markers in Erlo+GEM (G) and Erlo+DAC (H). I. Concentration-gradient response of Fer-1 in attenuating GP3-mediated ROS accumulation.

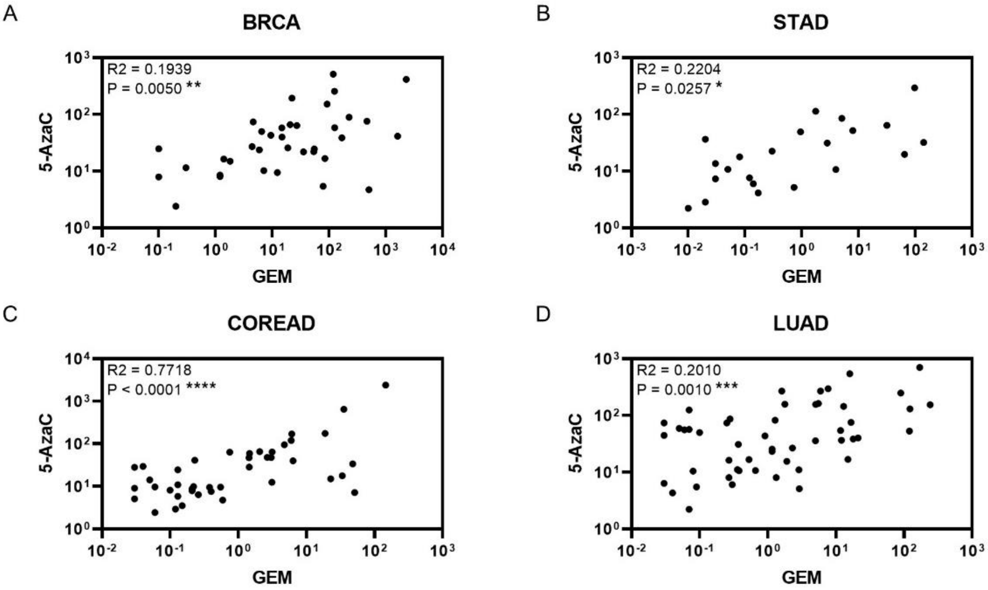

Fig. S5. Correlation of responses to Gemcitabine and 5-Azacytidine in multiple tumor cell lines. A-D. The IC50 values of Gemcitabine (GEM) and 5-Azacytidine (5-AzaC) in breast cancer (A), gastric cancer (B), colorectal adenocarcinoma (C), and lung adenocarcinoma (D) cell lines, as well as the correlation between their responses.

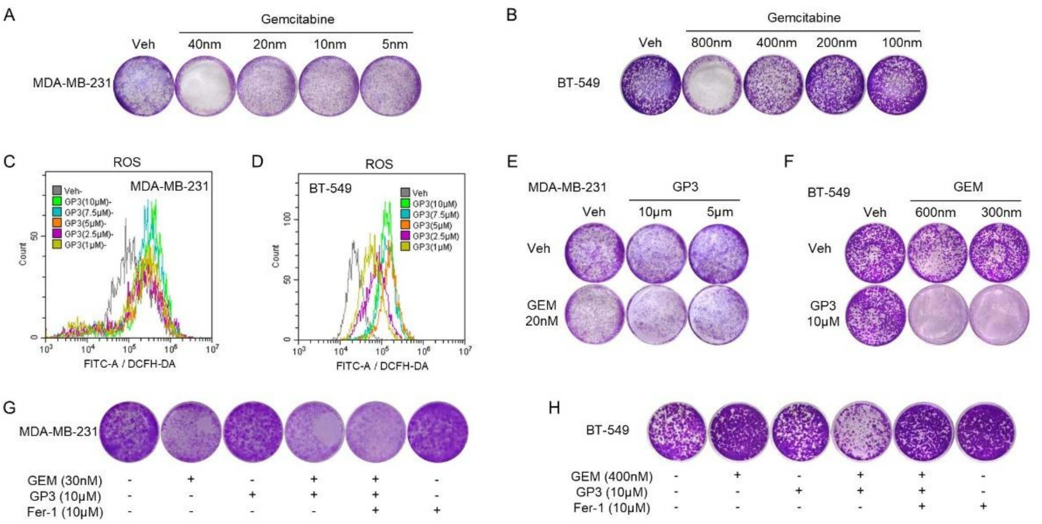

Fig. S6. The synergistic effects of GP3 and GEM in the human TNBR cell lines. A-B. Long-term inhibitory dose testing of GEM on MDA-MB-231 (A) and BT-549 (B). Treatment duration was 5 days. C-D. GP3-induced ROS in MDA-MB-231(C) and BT-549(D). E-F. The synergy effect of GP3 and GEM in MDA-MB-231(E) and BT-549(F). G-H. The effect of Fer-1 on GP3-induced ferroptosis susceptibility in MDA-MB-231 (G) and BT-549 (H) under GEM treatment.

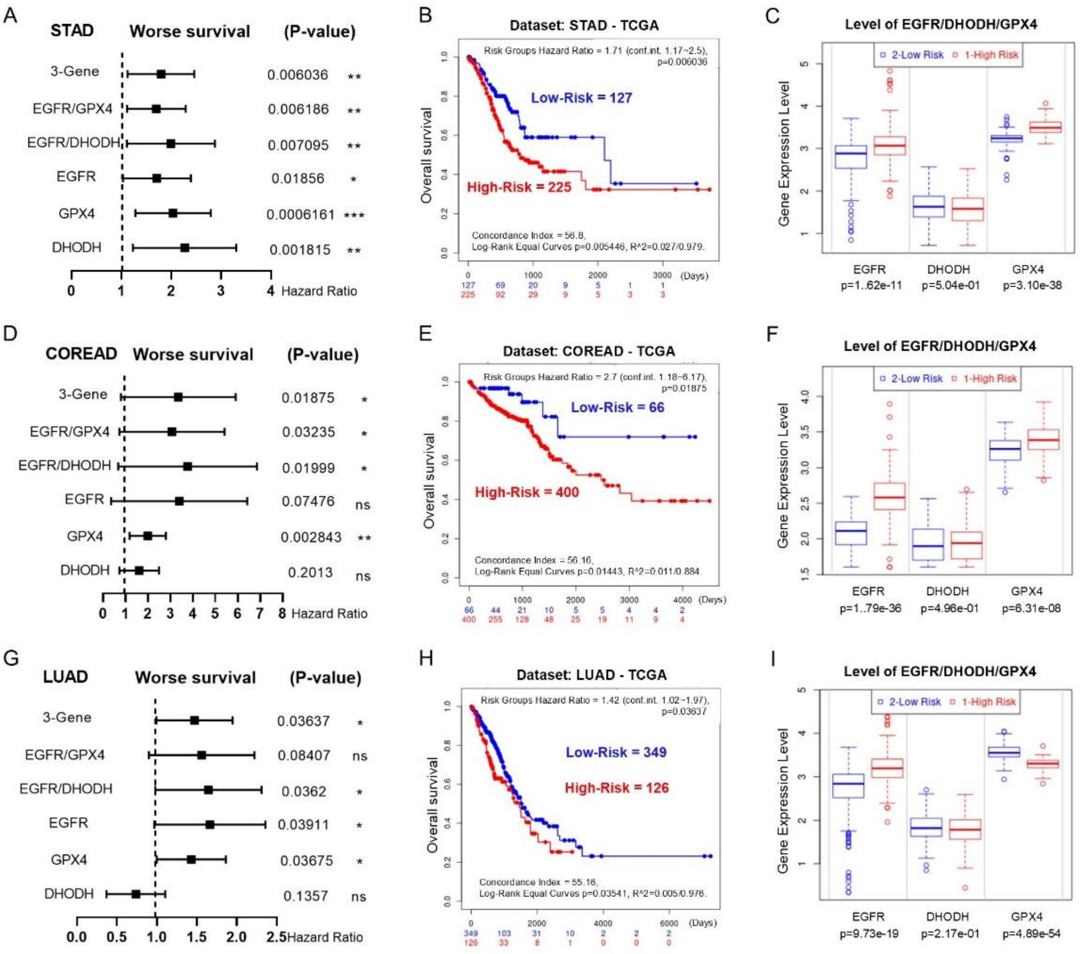

Fig. S7. Clinical relevance of DHODH, GPX4, EGFR expression. A-C. Forest plot for the prognostic performance of DHODH, GPX4, EGFR, and their combinations in TCGA-STAD (A), TCGA-COREAD (B), TCGA-LUAD (C). p-Value of the log-rank test were shown. Human cancer datasets (TCGA-STAD, n=352; TCGA-COREAD, n=467; TCGA-LUAD, n=475) were chosen to survival analysis. The hazard ratio (HR), confidence interval, p-Value in forest plot were obtained from the SurvExpress program. D-F. Survival curve of STAD (D), COREAD (E), LUAD (F) patients. G-I. the expression level of DHODH, GPX4, EGFR in low-risk and high-risk groups of TCGA-STAD (G), TCGA-COREAD (H), TCGA-LUAD (I). The prognostic index (PI) was calculated by the joint expression level of the 3 genes and the Cox model to generate the risk groups. The optimization algorithm was applied in risk grouping.

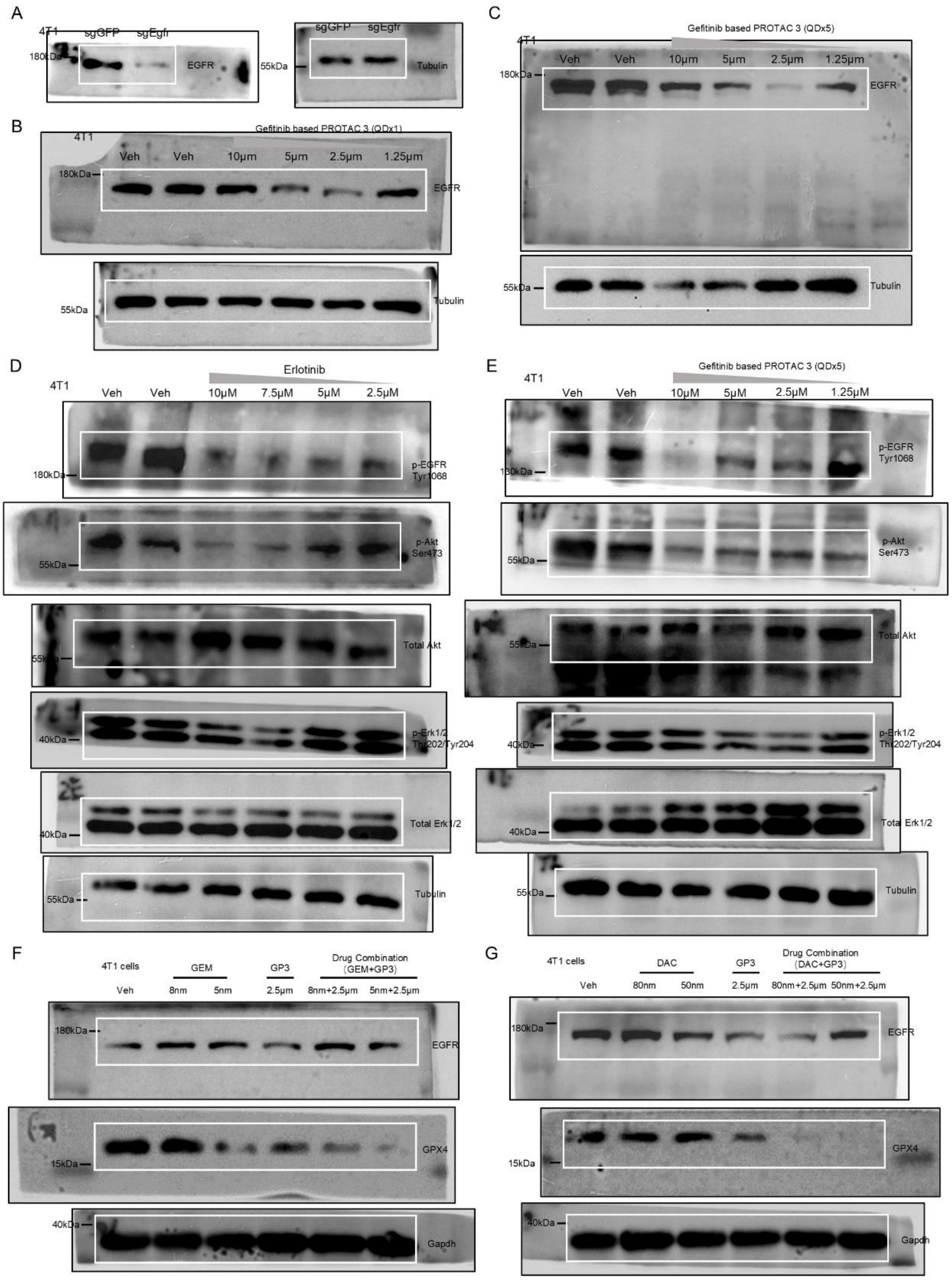

Fig. S8. Uncropped original Western Blot (WB) image. A. Related to Fig. 3E. B-C. Related to Fig. 4F-G. D-E. Related to Fig. 4H-I. F-G. Related to Fig. 7I-J.
